# New Solid Forms of Griseofulvin: A Solvate and a Relict Polymorph Related to Reported Solvates

**DOI:** 10.1021/acs.cgd.3c01011

**Published:** 2023-11-15

**Authors:** Mariana O. Diniz, Enrico Spoletti, Peuli Ghosh, Matteo Lusi, Michael Svärd, Åke Rasmuson, Sarah P. Hudson

**Affiliations:** † SSPC, the Science Foundation Ireland Research Centre for Pharmaceuticals, University of Limerick, Limerick V94 T9PX, Ireland; ‡ Department of Chemical Sciences, Bernal Institute, University of Limerick, Limerick V94 T9PX, Ireland; § Department of Chemical Engineering, KTH Royal Institute of Technology, Stockholm SE-100 44, Sweden

## Abstract

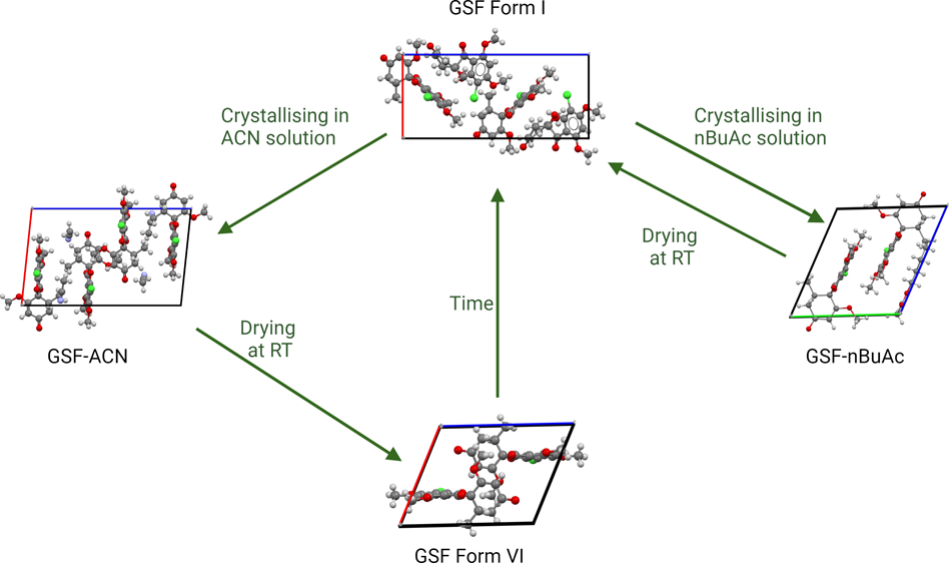

This work presents two new solid forms, a polymorph and a solvate, of the antifungal active pharmaceutical ingredient griseofulvin (GSF). The novel forms were characterized by powder X-ray diffraction, differential scanning calorimetry, and thermogravimetric analysis, and their crystal structures were determined by single-crystal X-ray diffraction. The new polymorphic form (GSF Form VI) was obtained upon drying at room temperature the GSF-acetonitrile solvate. GSF Form VI is a relict structure related to reported solvates of GSF. Thermal stability studies show that Form VI is metastable and monotropically related to the stable GSF Form I. The new GSF-*n*-butyl acetate solvate was obtained by crystallization from an *n*-butyl acetate solution. The stoichiometry of the *n*-butyl acetate solvate is 1:0.5. The solvate loses the solvent from the crystal lattice at a temperature between 363.15 and 374.15 K.

## Abstract 2

Schematic representation of griseofulvin new solid form transformations.

## Introduction

The landscape of possible solid forms of an active pharmaceutical ingredient (API) is important during its production, purification, and manufacture into a medicine. A solid form can either be crystalline, containing an ordered distribution of molecules or ions in a three-dimensional arrangement, or amorphous, lacking long-range order.^1,2^ The solid form of the final drug product can significantly affect its solubility, which is intrinsically related to its bioavailability. Upon administration to the body, the solid form influences the rate and extent of an API’s dissolution and, subsequently, its physiological absorption.^3^ Overall, the phenomenon of polymorphism is considered to be inherent to the crystalline state, and it has been stated that the number of polymorphic forms reported for a substance is proportional to the time spent searching for them.^4^

Dunitz and Bernstein (1995) described some of the possible consequences of poor crystal form screening, where well-characterized metastable forms could never be reproduced after the sudden isolation of a more stable form.^5^ In some instances, whole product formulations and industrial processes had to be revised, with dire consequences for patients. Bučar et al. (2015) revisited the issue, highlighting the importance of adequate control over nucleation and growth to enable the crystallization of the desired polymorphic form.^6^ Thus, mapping the landscape of crystalline forms is important to prevent the occurrence of unexpected solid forms during manufacture and/or storage.

**Figure 1:**
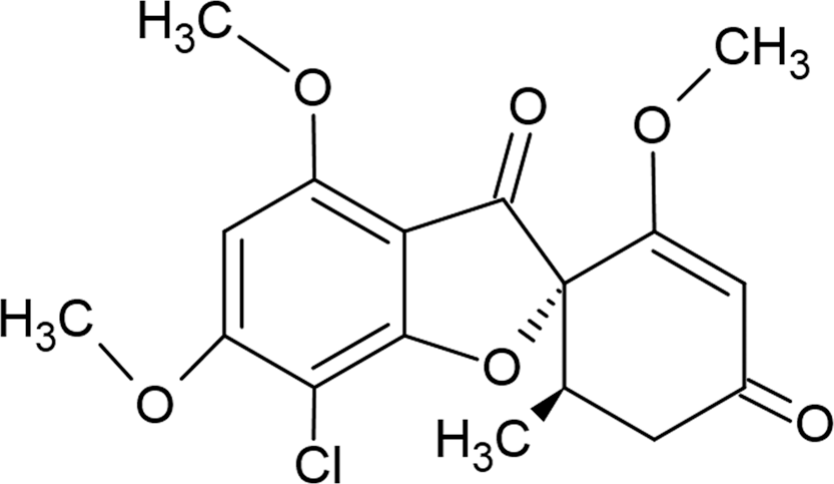
Chemical structure of griseofulvin.

Griseofulvin [C17H17ClO6-(2S,6’R)-7-chloro-2′,4,6-trimethoxy-6′-methyl-3H,4′H-spiro [1-benzo-furan-2,1′-cyclohex[2]ene]-3,4′-dione] (GSF) is an antifungal drug isolated from *Penicillium griseofulvum*.^7^ The drug is of wide interest as it is used to treat dermatomycoses such as ringworm, athlete’s foot, and infections of the scalp and nails.^8,9^ GSF is a class II drug according to the biopharmaceutics classification system (BCS), which means that it has low solubility and high permeability.^8^ The solid state of the API can play an important role in bioavailability, considering that metastable phases exhibit higher solubility than the stable form.^10−12^ The medicine is available as a tablet or suspension for oral administration as the stable Form I.^13^ The chemical structure of GSF, Figure 1, presents a benzofuran moiety combined with a cyclohexanone ring containing two chiral centers, two methoxy substituents on the benzofuran ring and one on the cyclohexanone ring, a carbonyl and a chloride substituent on the benzofuran moiety, and a methyl substituent on the cyclohexanone ring.

Such chemical and geometrical complexity translate into a rich polymorphic and solvate landscape for GSF. The first crystal structure of GSF (Form I, the stable polymorph) was reported in 1977.^14^ For many decades, GSF was considered to be monomorphic because all attempts to crystallize it from solution resulted in the formation of Form I, despite variation of crystallization protocols. However, different solvates have been reported: with chloroform,^15^ benzene, dioxane,^16^ bromoethane, dichloroethane, dichloromethane, dibromomethane, bromochloromethane, 1-bromo-2-chloroethane,^17^ acetonitrile, nitromethane, and nitroethane.^8,18^ In 2013, two new GSF polymorphs (Form II and Form III) were obtained by melt crystallization.^19^ The respective crystal structures were solved in 2018 (Form II)^20^ and 2020 (Form III).^21^ In 2022, two additional polymorphs (Form IV and Form V) were obtained from the melt in the presence of PEG.^22^

In this work, we report a new polymorphic form (GSF Form VI) as well as a new solvate form (GSF-*n*BuAc) of GSF. The new polymorph was obtained upon desolvation of the known GSF-acetonitrile (GSF-ACN) solvate at room temperature (RT).^8^ The new solvate was obtained from a GSF solution in *n*-butyl acetate (*n*BuAc). In-depth structure analysis of these new solid forms and previously published solvates indicates that this novel GSF Form VI is a relict metastable structure related to GSF solvates shedding light on the solvate desolvation process. The discovery of these new solid forms of GSF highlights the importance of fully mapping the crystallization landscape, demonstrating that the discovery of additional forms of established drug APIs is clearly feasible.

## Experimental Section

### Materials

Griseofulvin (GSF) Form I (98%) was obtained from Baoji Guokang Bio-Technology Co. (China), and the solvents acetonitrile (ACN) (HPLC gradient grade) and *n*-butyl acetate (*n*BuAc) (99+%) were obtained from Fisher Scientific. All chemicals were used without further purification.

### Powder X-ray Diffraction (PXRD)

Powder samples were obtained by cooling crystallization. Undersaturated solutions of GSF in ACN within the concentration range 37–45 g L^–1^ were stirred at 313.15 K for 72 h and filtered using nylon 0.2 μm filters. Undersaturated solutions of GSF in *n*BuAc within the concentration range 8–10 g L^–1^ were stirred at 333.15 K for 72 h and filtered using polytetrafluoroethylene (PTFE) 0.2 μm filters. The resulting solutions in both solvents were left to crystallize at 283.15 K and 1200 rpm in sealed vials. This temperature was selected to generate a range of supersaturations, calculated as the ratio between the solution concentration and solubility of GSF Form I in the respective solvent, as reported by Zhao et al.^23^ The supersaturation range for GSF was 1.41–1.86 in ACN and 2.13–2.57 in *n*BuAc. The crystallization of GSF in the supersaturated solutions could be visibly detected by the transition from a clear solution to a cloudy one. The white suspensions obtained by crystallization were then filtered using 0.22 μm polyvinylidene fluoride (PVDF) membrane filters and left to dry at RT in a fume hood. The slurry containing GSF-ACN solvate and freshly filtered samples of GSF-*n*BuAc solvate powder was analyzed by PXRD analysis. PXRD data were collected in reflection mode with an Empyrean diffractometer (PANalytical, Phillips) equipped with Cu Kα radiation (λ = 1.5406 Å) operating at 45 kV and 40 mA at RT. Samples were scanned between 2θ values of 5 and 45° at a step size of 0.01313° and at 18.87 s per step.

### Synthesis and Characterization of Single Crystals

Undersaturated solutions of GSF in ACN within the concentration range 37–45 g L^–1^ and in *n*BuAc within the concentration range 8 - 10 g L^–1^ were stirred for 72 h at 313.15 and 333.15 K, respectively. The resulting solution was then placed in open glass Petri dishes at RT inside a fume hood. Crystals formed upon complete solvent evaporation were collected for analysis. A small amount of sample was dispersed in perfluoroether oil on a glass slide and inspected under cross-polarized light on a microscope. A suitable single crystal was selected, collected using a fiber loop, and analyzed by single-crystal X-ray diffraction (SC-XRD). The crystal structure was determined at RT and at 150 K for crystals obtained from ACN solution, and at 150 K for the crystals obtained from *n*BuAc solution, by X-ray diffraction on a Bruker D8 Quest diffractometer equipped either with a Cu Kα (λ = 1.54178 Å) radiation or Mo Kα (λ = 0.71073 Å) radiation and Photon 100 detector. The data were integrated with the Bruker SC-XRD software Apex 4. The structures were solved by the intrinsic phasing methods and refined by least-squares methods against *F*^2^_obbs_ using SHELXT^24^ and SHELXL^25^ with the OLEX2^26^ interface. Non-hydrogen atoms were refined anisotropically. Hydrogen atoms were placed in calculated positions using standard riding model constraints and refined isotropically with Uiso fixed at 1.2 times the one of the parent atom (1.5 for methyl groups). The software Mercury 2022.3.0 was used for graphic representations.^27^

### Stability Evaluation

Samples of crystals obtained in ACN and in *n*BuAc solutions were incubated overnight (∼ 20 h) at 423.15 and 323.15 K, respectively. The PVDF membrane filter containing the solids after filtration was placed in a petri dish and left open in a clean oven set at the desired temperature. The resulting powder samples were analyzed by PXRD to identify structural transitions.

### Thermal Analysis

Thermogravimetric analysis (TGA) was carried out under a nitrogen flow of 20 mL min^–1^ using a Perkin-Elmer TGA 4000. Samples containing 2–5 mg of powder were placed in a ceramic crucible and heated to 523.15 K at a ramp rate of 20 K min^–1^.

Differential scanning calorimetry (DSC) was performed using a Netsch Polyma 214 DSC. The furnace cell was precalibrated against the melting properties of 5 model materials. 2–4 mg of powder was added to a concavus aluminum pan, which was sealed using a crimping press, and then the lid was pierced to avoid an increase in pressure due to sample evaporation. The samples were analyzed in an inert environment (nitrogen flow of 20 mL min^–1^) with a temperature ramp rate of 5 K min^–1^ from 293.15 to 533.15 K.

### Crystal Habit Analysis

The crystal habit of the powder samples of new GSF forms was characterized using a HITACHI SU-70 (Hitachi Inc., Japan) scanning electron microscope (SEM) instrument. A small amount of the powder was placed onto an adhesive carbon tape previously attached to a cylindrical aluminum 15 mm SEM stub. The samples were coated with gold using an Emitech K550 (Emitech, United Kingdom) sputter coater at 20 mA for 60 s. The particles were imaged at a voltage of 3–10 kV. All SEM images show particles which were fully representative of the entire sample analyzed in each case.

The crystal habit of crystals formed by solvent evaporation was characterized using an Olympus BX51 polarized light optical microscope (PLM). A few drops of undersaturated solutions of GSF in ACN and GSF in *n*BuAc were placed on a glass slide. During solvent evaporation, microscope images were collected as freshly crystallized after 24 h drying at RT and after incubating for 1 h at 423 K.

### Fourier Transform Infrared (FTIR) Spectroscopy

The infrared spectra were collected for GSF Form I and VI powders and a slurry of GSF-*n*BuAc using a Thermo Fisher Scientific Nicolet iS50 infrared spectrometer with an attenuated total reflection (ATR) unit and collecting program, OMINIC. Spectra were recorded between 400 and 4000 cm^–1^ using 8 cm^–1^ spectral resolution and 64 scans per sample.

### Hirshfeld Surface Analysis, Energy Frameworks, and Lattice Energies of Crystal Structures

The intermolecular interactions within the GSF crystal structures were identified using a molecular Hirshfeld surface and fingerprint analysis and the plots were generated using the software CrystalExplorer21.^28^ The crystallographic data for GSF Form I (CSD Refcode GRISFL07^20^) and Form VI (this work) were used as structural models. Additionally, a hypothetical structure was simulated by deleting the solvent molecules from the GSF-*n*BuAc model structure. Interaction and lattice energies were calculated for 3.8 and 20 Å cluster of molecules, respectively, using B3LYP/6–31G(d,p) wave function. The interaction energies were graphically represented as a framework by linking the centers of mass of molecules with cylinder thickness, corresponding to the total intermolecular interaction energies. Interaction energies less than 10 kJ mol^–1^ were omitted for clarity.

## Results and Discussion

### PXRD Analysis of GSF Form VI and GSF-*n*BuAc Solvate

**Figure 2:**
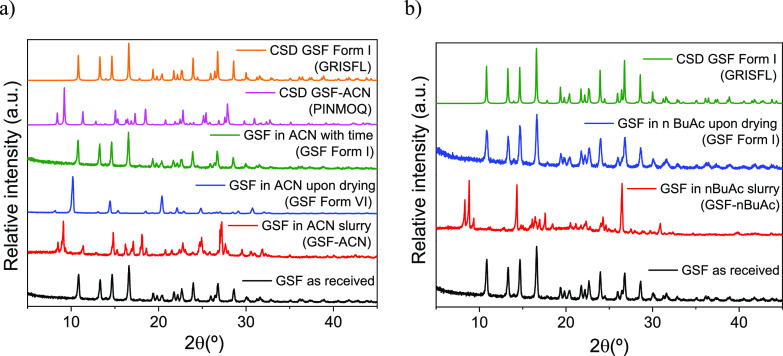
PXRD pattern of the solid-state changes in the GSF crystals formed in (a) ACN and (b) *n*BuAc.

When GSF Form I, as received, is dissolved in ACN solution, it recrystallizes as the GSF-ACN solvate (Figure 2a).^8^ Upon drying at ambient temperature (around 291–298 K) for 24 h, a novel phase crystallized whose structure was not previously present in the CSD database, henceforth called Form VI (Figure 2a). GSF Form VI transforms into the stable Form I after six months if kept in a closed vial at RT (291–298 K) or in less time if exposed to high relative air humidity (Figure 2a). The exposure of GSF-ACN crystals to air at RT (291–298 K) for 24 h provides single crystals of the novel phase (GSF Form VI) suitable for SC-XRD analysis. The new polymorph (GSF form VI) crystallizes in the monoclinic *P*2_1_ space group, with *a* = 9.4957 Å, *b* = 8.6772 Å, *c* = 11.8614 Å, and β = 113.465° (Table 1). The unit cell contains 2 GSF molecules (*Z* = 2), with one molecule in the asymmetric unit. The crystal structures of the reported forms of GSF are presented in Table S1 in the Supporting Information.

**Table 1:** Crystal Data and Details of Measurements for GSF Form VI and GSF-*n*BuAc Solvate

	GSF Form VI	GSF Form VI	GSF-*n*BuAc solvate
chemical formula	C_17_H_17_ClO_6_	C_17_H_17_ClO_6_	C_17_H_17_ClO_6_·0.5C_6_H_12_O_2_
*M*_w_, g mol^–1^	352.75	352.75	410.83
*T*/K	298.00	150.00	150.00
radiation source	copper	copper	molybdenum
crystal system	monoclinic	monoclinic	triclinic
space group	*P*2_1_	*P*2_1_	*P*1
*a*/Å	9.4957(4)	9.3824(6)	8.6087(3)
*b*/Å	8.6772(4)	8.6221(5)	11.7252(4)
*c*/Å	11.8614(5)	11.8304(7)	11.7453(4)
α/°	90	90	113.2290(10)
β/°	113.465(2)	113.240(3)	90.0600(10)
γ/°	90	90	110.9930(10)
*V*/Å^3^	896.51(7)	879.38(9)	1003.04(6)
Z, Z′	2,1	2,1	2,2
*d*/g cm^–3^	1.307	1.332	1.360
μ/mm^–1^	2.142	2.184	0.229
measured reflections	18884	30280	39116
independent reflections	3268	3176	11798
Reflections with *I*> 2σ(*I*)	2783	2809	9185
*R*_int_	0.0561	0.0747	0.0482
*R*[*F*^2^> 2σ(*F*^2^)]	0.0408	0.0550	0.0494
*wR*_2_(*F*^2^)	0.1093	0.1497	0.1285

Similarly, PXRD analysis suggests that the recrystallization of GSF Form I from *n*BuAc solution affords a new GSF-*n*BuAc solvate (Figure 2b). Upon drying at RT for 24 h, GSF-*n*BuAc undergoes desolvation and converts into the stable GSF Form I (Figure 2b). Single crystals of the new solvate, at 150 K, adopt a triclinic space group, with *a* = 8.6087 Å, *b* = 11.7252 Å, *c* = 11.7453 Å, α = 113.229°, β = 90.060°, and γ = 110.993° (Table 1). The unit cell contains 2 molecules of GSF and 1 *n*BuAc in the asymmetric unit. The crystal structures of reported solvated forms of GSF are presented in Table S2 in the Supporting Information. The crystal is isostructural to that of GSF-dichloromethane^17,18^ as well as isometric to other GSF solvates (-bromochloromethane, -dibromomethane and -bromoethane^17^), whose full structures were never reported but whose crystal systems are reported in the CSD.

**Figure 3:**
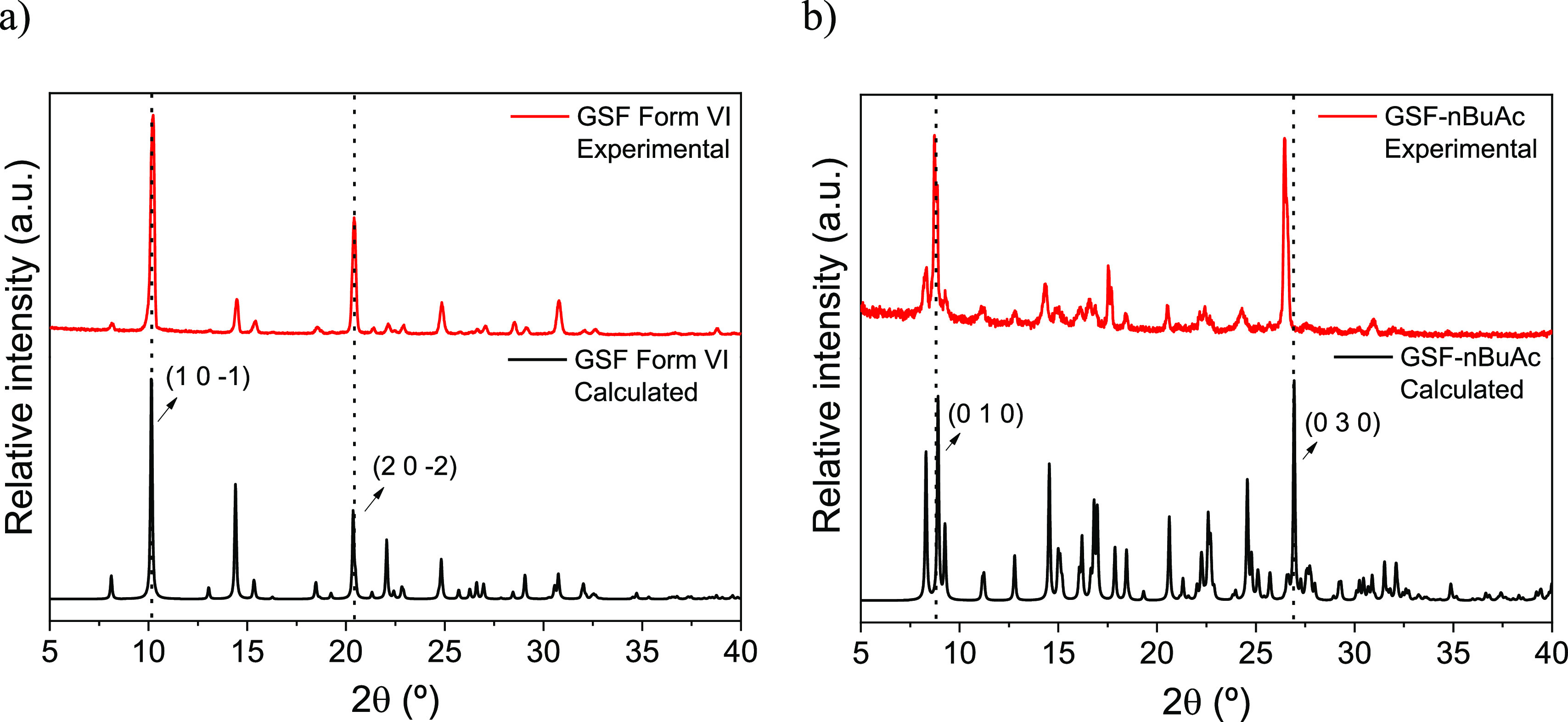
PXRD pattern calculated and experimental for (a) GSF Form VI and (b) GSF-*n*BuAc.

The PXRD of bulk Form VI indicates a substantial correspondence with the pattern calculated from the single-crystal data (Figure 3a). Minor differences in peak intensities are compatible with preferred orientation effects. The experimental PXRD pattern for GSF-*n*BuAc is also superimposable onto the calculated ones from the single-crystal model (Figure 3b), although, in this case, peak broadness suggests a lower crystallinity of the bulk. In addition, a peak shift is noticeable, which is a consequence of thermal expansion. In particular, the (030) peak shifts to higher angles (shorter d-spacing) upon cooling.

### Crystal Structure Analysis of the New GSF Solid Forms

**Figure 4:**
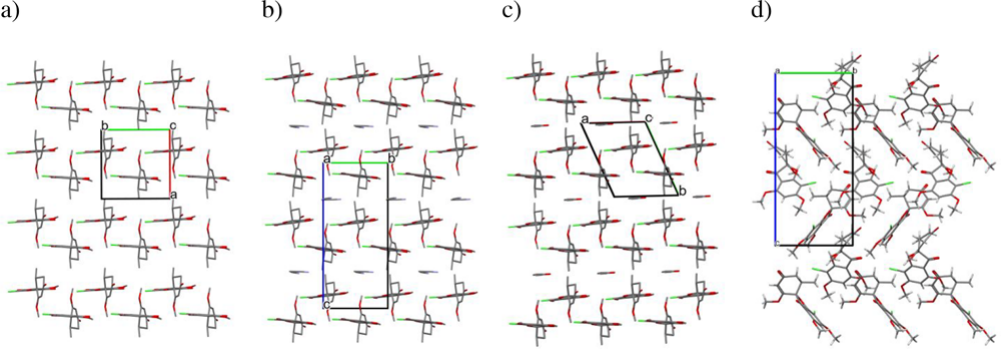
Crystal packing of (a) GSF Form VI, (b) GSF-ACN solvate, (c) GSF-*n*BuAc, and (d) GSF Form I.

**Figure 5:**
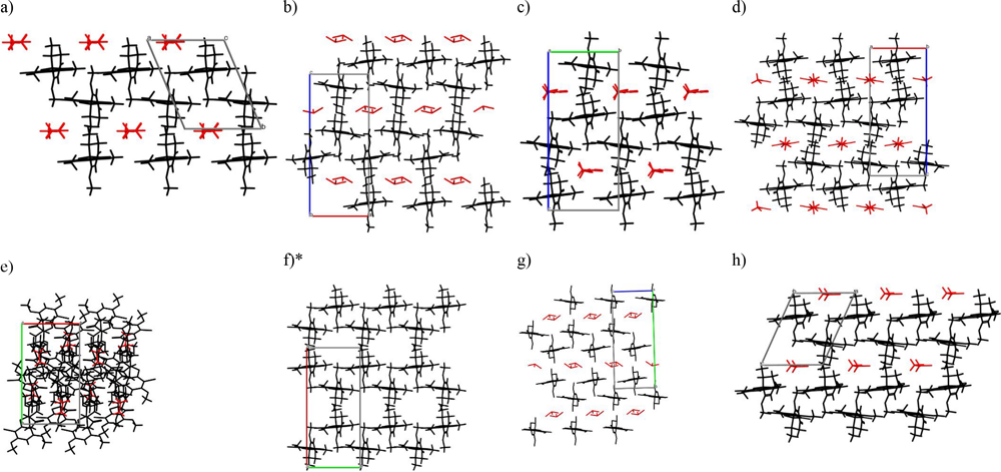
Crystal structure of solvated forms of GSF containing voids (a) GSF-*n*BuAc, (b) GSF – chloroform, (c) GSF – acetonitrile, (d) GSF – nitromethane, (e) GSF – nitroethane (2:1), (f) GSF – nitroethane (1:1), (g) GSF-dichloroethane, and (h) GSF – dichloromethane. GSF molecules are represented in black and solvent molecules are represented in red. The unit cells of the GSF solvates not shown are missing in the CSD database. *Disordered solvent molecules were not modeled by the authors.

The crystal structures of GSF Form VI, GSF-ACN, and GSF-*n*BuAc are characterized by π-stacked double layers of GSF molecules. Within each strand of the double layer, molecules are held together by a combination of Cl–H and Cl–O interactions and are oriented in the same direction. In Form VI and in the ACN solvate, the double layers extend along the crystallographic screw axis, Figure 4; the triclinic *n*BuAc solvate lacks the screw axis but a similar structure is observed. As depicted in Figure 4, along the vertical direction, all the structures have the same periodicity, and the differences in the unit cell metrics are due to changes in packing symmetry and the number of independent molecules. However, the double layers of Form VI repeat in an ab–ab fashion, whereas in the solvates they are shifted with respect to one another, resulting in an ab–ba repetition. The latter creates empty space that can accommodate the solvent molecules. Such structural similarity, together with the observed synthetic conditions, suggests that GSF Form VI could be a relict structure that emerges from the removal of the guest solvent upon collapsing of the voids. Aitipamula et al. (2014) observed solvents occupying voids in the GSF-ACN, GSF- nitromethane (1:1), and GSF- nitroethane (1:1) solvates^8^ and Chen et al. (2019) had proposed a similar theoretical structure for the GSF-dichloromethane solvate.^18^ Indeed, all of the crystal structures of GSF solvates, with the exception of the GSF-nitroethane (2:1), present the same layered structure with voids occupied by different solvent molecules, Figure 5.

### Hirshfeld Surfaces, Energy Frameworks, and Lattice Energies of Crystal Structures

**Figure 6:**
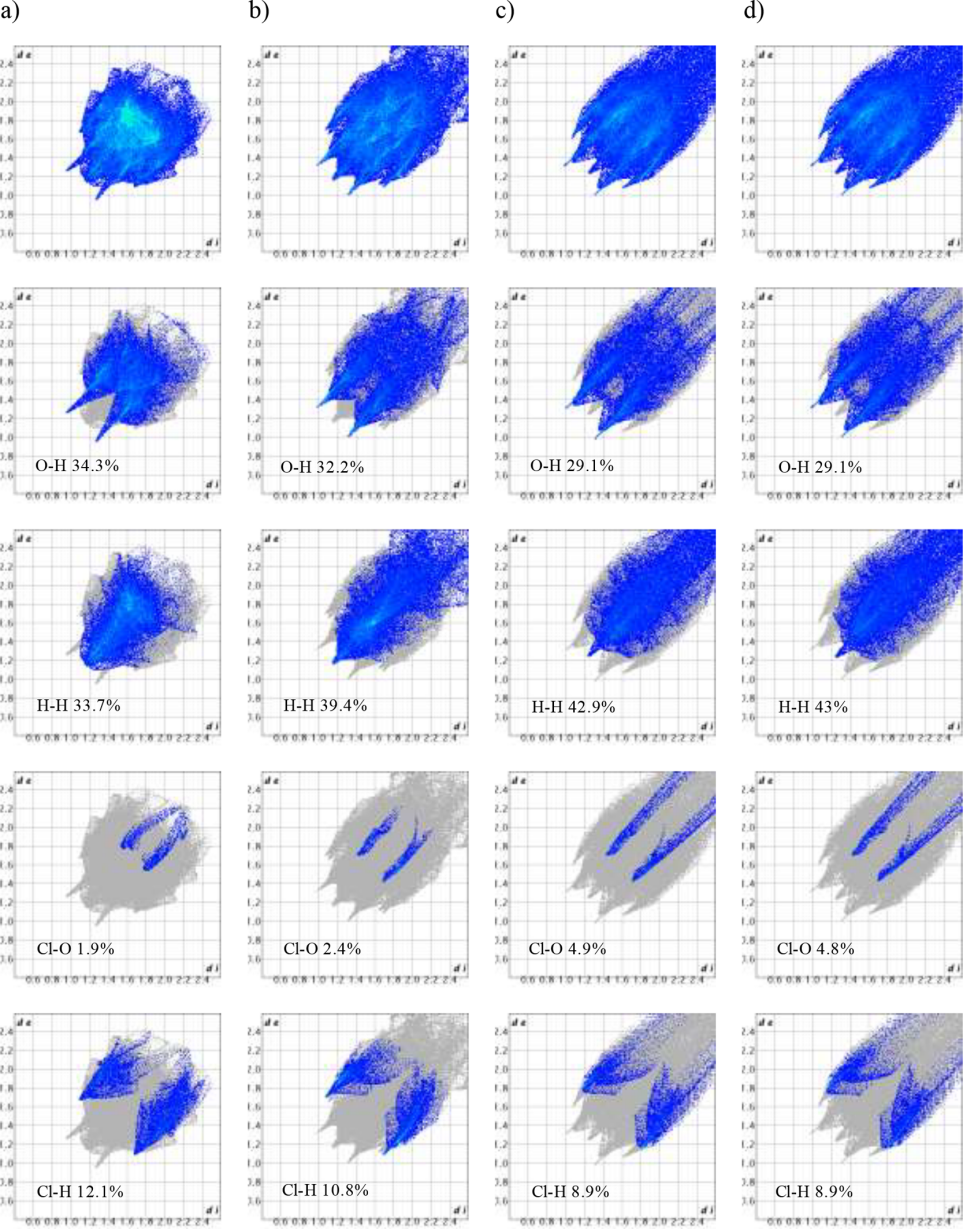
Hirshfeld fingerprint plots for (a) GSF Form I, (b) GSF Form VI, (c) GSF-*n*BuAc desolvated molecule a, and (d) GSF-*n*BuAc desolvated molecule b.

The Hirshfeld 2-D fingerprint surfaces have been plotted to highlight the different intermolecular interactions in the crystal structure of GSF Form I, GSF Form VI, and a pseudostructure containing two molecules in the asymmetric unit (molecules a and b), obtained by omitting the solvent from the model of GSF-*n*BuAc, Figure 6. The 2-D plot for GSF Form I shows a denser (more efficient) packing with no interactions in the top right quadrant, with the molecular volume of GSF Form VI (448.25 Å^3^) being larger than that of GSF Form I (399.48 Å^3^). H-bonds (O–H) in GSF Form I are the shortest of the series and the dispersive H–H contacts are longer than in Form VI. The new polymorph instead shows longer (i.e., possibly weaker) O–H contacts. Additionally Form VI has a more extended Cl–O contact (2.4 vs 1.9%) and narrower Cl–H interactions (10.8 vs 12.1%) of the total Hirshfeld surface. Overall, such differences justify the relative stability of the two polymorphs. The surface for the framework of the desolvated GSF-*n*BuAc is somewhat in between the two extremes, with more relaxed O–H and H–H contacts.

**Figure 7:**
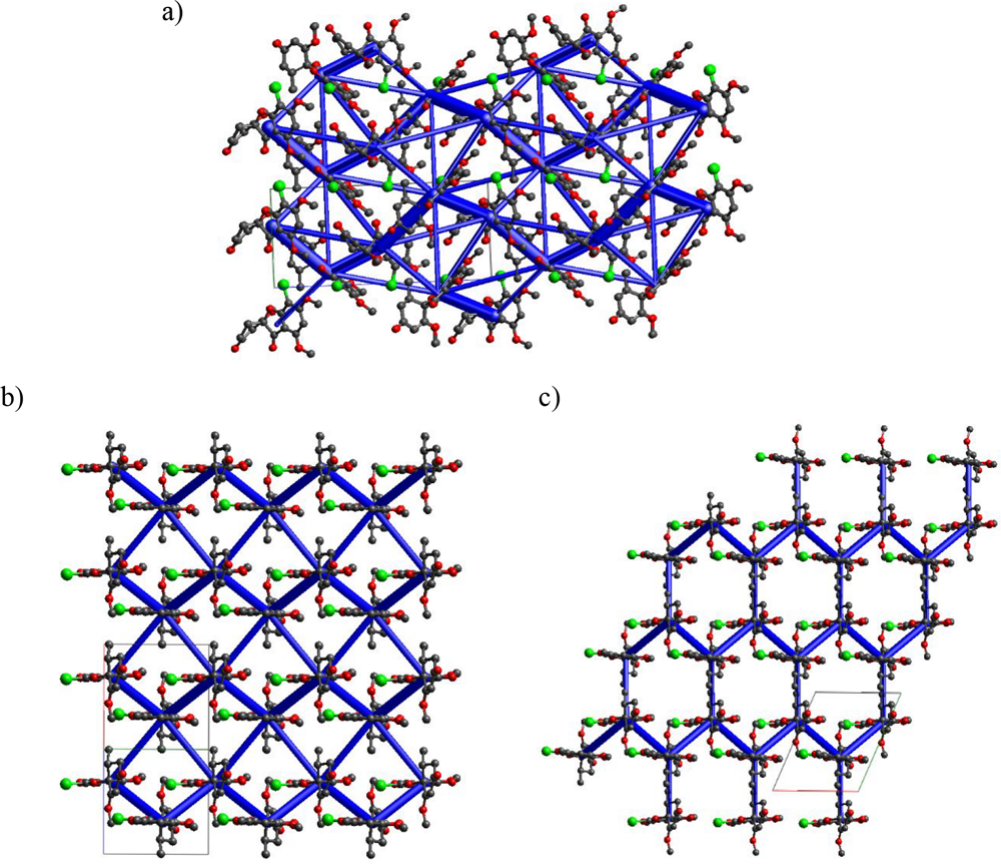
Energy frameworks of (a) GSF Form I, (b) GSF Form VI, and (c) theoretical desolvated GSF-*n*BuAc. The energy threshold for the energy framework is set at 10 kJ mol^–1^.

The energy frameworks for GSF Form I, Form VI, and the pseudo desolvated GSF-*n*BuAc are presented in Figure 7. The energy framework of GSF Form I indicates that the molecules are closely packed and held together by a complex pattern of interactions. In the GSF Form VI, the lattice presents a network with strong zigzag interactions within the double layers and similar but weaker (dispersive) interactions across successive double layers. The desolvated GSF-*n*BuAc form shows a honeycomb energy framework with a zigzag motif within the double layer but much weaker, perpendicular, and elongated across successive layers. The anisotropic arrangement of weak and strong interactions could explain why the structure of the solvates collapse upon guest removal while the double layers are preserved. Indeed, GSF Form VI was only isolated from GSF-ACN, though it might occur, in nondetectable traces, from other isostructural solvates. Furthermore, the observed zigzag framework has been associated with increased crystal plasticity.^32,33^ Previously, Chen et al. (2019) reported improved compressibility in GSF powder granulated with DCM and attributed the improvement to the formation of a porous phase.^18^ Here, instead, the cause of the reported increased compressibility may be the presence of metastable GSF Form VI.

The lattice energies calculated for GSF Form I, Form VI, and GSF-*n*BuAc desolvated are −41.9, −35.9, and −32.6 kcal mol^–1^, respectively. Therefore, GSF Form I has closer and stronger intermolecular interactions that stabilize the crystal structure. The virtual GSF-*n*BuAc desolvated form was too unstable to be isolated. GSF Form VI is a metastable form that can be isolated after desolvation of the GSF-ACN solvate and presents a structure similar to the other GSF solvates containing voids such as the GSF-*n*BuAc. Intermolecular and lattice energies of the new solid forms of GSF are presented in Tables S3–S10 in the Supporting Information.

### Thermal Analysis

**Figure 8:**
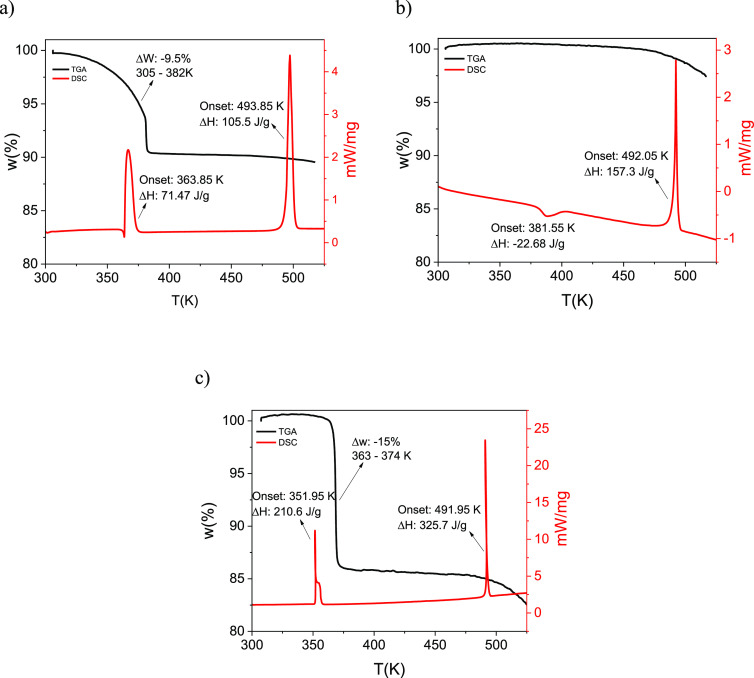
TGA and DSC of (a) GSF-ACN, (b) GSF Form VI, and (c) GSF-*n*BuAc solvate.

The TGA profile and DSC thermogram of GSF-ACN (Figure 8a) agree with the data that has previously been reported by Aitipamula et al.,^8^ confirming it has a 1:1 stoichiometric ratio. The DSC shows one endothermic peak at 363.85 K corresponding to the desolvation of the solvate and another endothermic peak at 493.85 K corresponding to the melting of GSF Form I, but the transition of the solvate to GSF Form VI and then to Form I was not detected during the DSC analysis.

The DSC thermogram for GSF Form VI, Figure 8b, shows an exothermic peak at 381.55 K corresponding to the transformation into GSF Form I, and an endothermic peak at 492.05 K corresponding to the melting of GSF Form I. According to the heat of transition rule proposed by Burger and Ramberger (1979), if an endothermic peak is observed in the DSC thermogram, it indicates a transition between two forms that are related enantiotropically, while if an exothermic peak is observed, it indicates a transformation between two forms that are related monotropically.^34,35^ Based on this rule, the DSC result suggests a monotropic polymorphic relationship between the previously unreported GSF Form VI and Form I. The TGA profiles do not show weight loss during the transformation from Form VI into Form I, indicating that Form VI is formed after a full desolvation of the GSF-ACN solvate. At higher temperatures, there is a gradual loss of mass with an unclear onset temperature above 471.15 K due to the melt-induced thermal decomposition of the GSF.

The TGA profile and DSC thermogram of the GSF-*n*BuAc solvate are presented in Figure 8c. The TGA profile presents a sharp loss of mass (15%) occurring between 363.15 and 374.15 K, corresponding to the solvent completely leaving the crystal structure. The stoichiometry of the GSF-*n*BuAc solvate is two molecules of GSF for every molecule of *n*BuAc (GSF:*n*BuAc – 1:0.5, which correlates to 14.2% solvent content by mass), corresponding to the crystal structure determination. The DSC thermogram shows an endothermic peak at 351.95 K corresponding to the collapse of the solvate structure and recrystallization into GSF Form I and evaporation of the solvent molecules^19^ and an endothermic peak at 491.95 K corresponding to the melting of the stable Form I.

### Stability Evaluation

**Figure 9:**
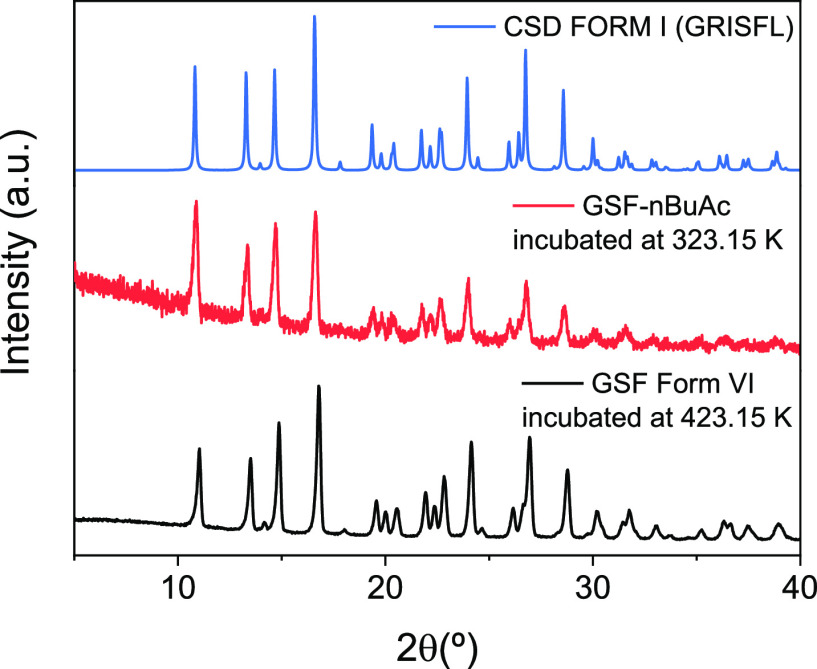
PXRD pattern of thermal stability tests for GSF Form VI and GSF-*n*BuAc.

PXRD analysis of the solid samples of GSF Form VI and GSF-*n*BuAc incubated for around 20 h at 423.15 and 323.15 K, respectively, are presented in Figure 9. The PXRD patterns show that both GSF Form VI and GSF-*n*BuAc completely transformed into Form I. The GSF-*n*BuAc transformation into GSF Form I at this temperature, lower than the transformation indicated by DSC, was due to a longer heating time (∼20 h). This clearly shows that the desolvated forms are metastable at these temperatures and undergo transformation to the most stable form.

### FTIR Analysis

**Figure 10:**
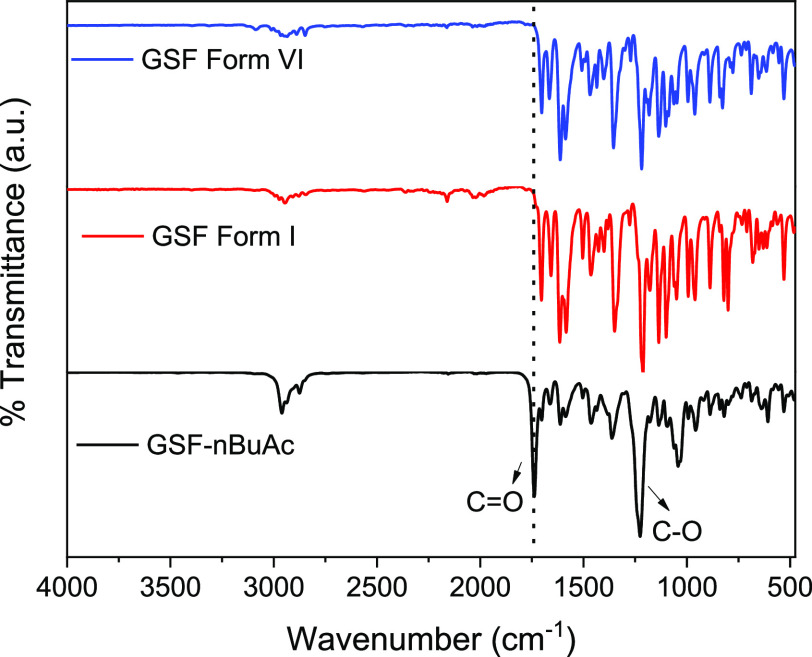
FTIR spectra for GSF Forms I and VI and GSF-*n*BuAc.

The FTIR spectra of GSF Forms I and VI and GSF-*n*BuAc present a medium shoulder at 2840–3000 cm^–1^ corresponding to the C–H alkane stretching (with the peak observed for the solvate in this region being more intense due to the butane solvate molecules present), peaks at 1705 cm^–1^ corresponding to the C=O aliphatic ketone, peaks at 1600–1670 cm^–1^ corresponding to alkene C=C stretching, peaks at 1450 cm^–1^ corresponding to the C–H bending in the methyl group, peaks at 1350 cm^–1^ corresponding to the C–O stretching in the alkyl aryl ether, peaks at 1205 cm^–1^ corresponding to the C–O stretching vinyl ether, and peaks at 600 cm^–1^ corresponding to the C–Cl stretching (Figure 10). These peaks are characteristic of the GSF molecular structure. The FTIR for GSF-*n*BuAc solvate presents two strong peaks at 1735 and 1220 cm^–1^ corresponding to the C=O and C–O stretching of the ester group in *n*BuAc, respectively. The FTIR spectra for GSF show no significant differences between the two polymorphic forms, and two extra peaks corresponding to the solvent groups were present in the spectrum of the GSF-*n*BuAc solvate.

### Crystal Habit Analysis

**Figure 11:**
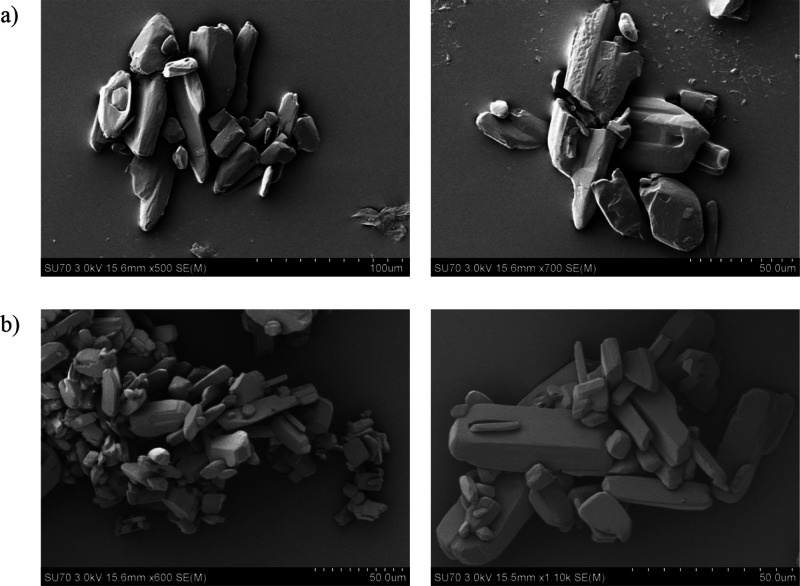
SEM images of (a) GSF Form VI and (b) GSF-*n*BuAc.

The SEM images of powder samples of GSF Form VI and GSF-*n*BuAc obtained by cooling crystallization are presented in Figure 11. GSF Form VI sample crystals present an irregular habit of nonhomogeneous size. GSF-*n*BuAc crystals present blocky shapes with different sizes.

**Figure 12:**
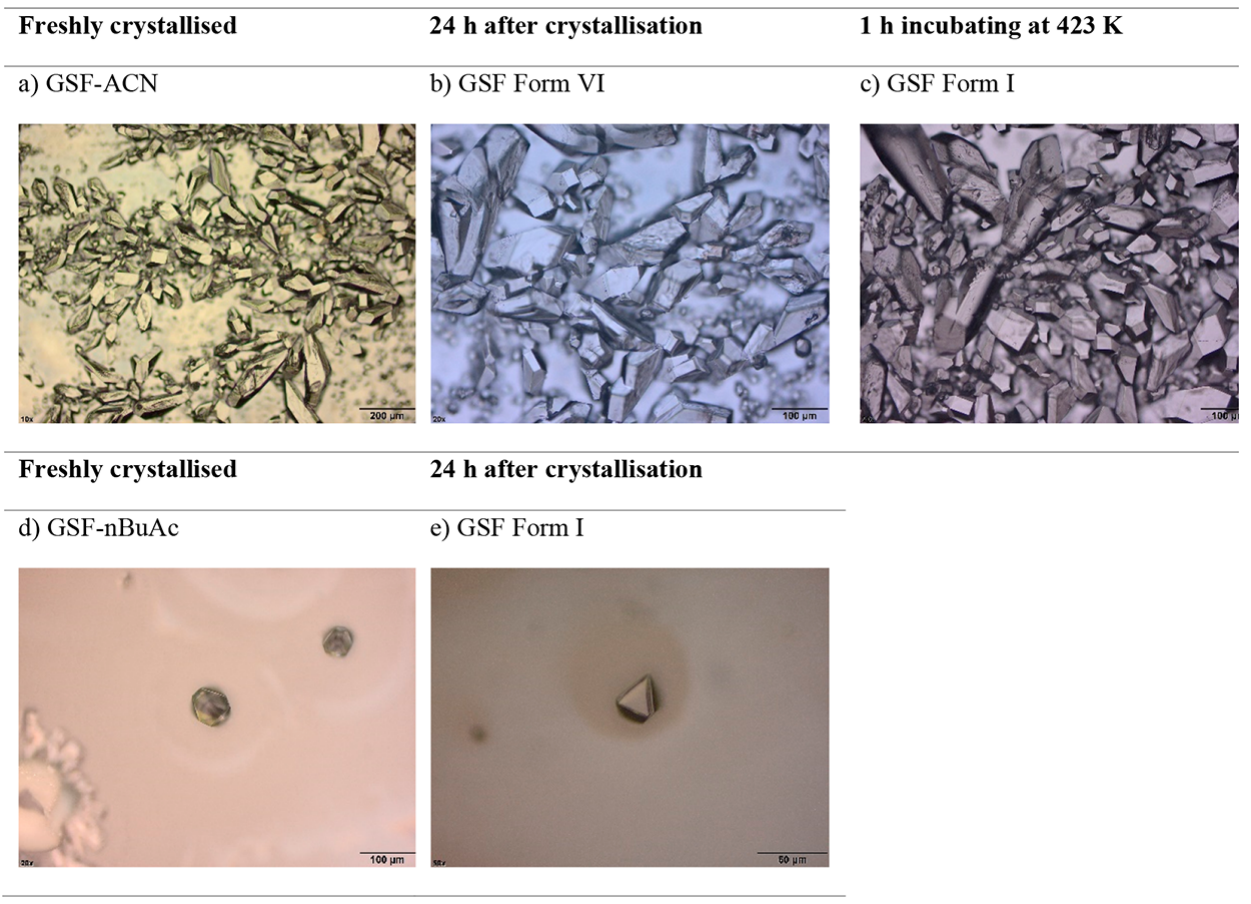
Optical microscopy image of crystals obtained by solvent evaporation of GSF-ACN solution: (a) GSF-ACN, (b) GSF Form VI, (c) GSF Form I, and of GSF-*n*BuAc solution: (d) GSF-*n*BuAc, (e) GSF Form I.

Optical microscope images of GSF-ACN, GSF Form VI, GSF-*n*BuAc, and GSF Form I obtained by solvent evaporation crystallization are shown in Figure 12. Based on the solid-state analysis presented in this work, GSF crystallizes as the GSF-ACN solvate in ACN and transforms to GSF Form VI after RT exposure for 24 h and to GSF Form I after heating at 423 K; and GSF crystallizes as GSF-*n*BuAc in butyl acetate and transforms to GSF Form I after drying for 24 h. Therefore, the microscope images obtained from the solvates after solvent evaporation and presented in Figure 12a–c correspond to (a) GSF-ACN solvate, (b) GSF Form VI, and finally (c) GSF Form I. In addition, the microscope image in Figure 12d corresponds to GSF-*n*BuAc, and in Figure 12e, it corresponds to Form I. GSF crystallized in ACN present platelike shapes, while GSF crystallized in *n*BuAc present diamond-like shapes. Therefore, the habit of the GSF crystals was affected by the solvent employed during the crystallization. However, the transformation from GSF-ACN to GSF Form VI and from GSF-*n*BuAc to GSF Form I does not affect the resulting crystal habit. As expected, given that the crystals presented in Figures 11 and 12 were crystallized by different methods under different conditions, the habit of the GSF crystals differs.

## Conclusions

A new polymorphic form and a new solvate form of GSF have been discovered, and their structures have been determined. GSF-*n*BuAc is a hemisolvate analogue of other solvates reported for GSF. GSF-*n*BuAc converts to GSF Form I upon heating to 323.15 K or by exposure to ambient air. GSF Form VI emerges as a metastable relict structure following the desolvation of the GSF-ACN solvate. GSF Form VI is metastable, transforming into Form I upon aging at RT or by incubating at 423.15 K. DSC analysis indicates that GSF Form VI is monotropically related to Form I. GSF Form VI might be the reason for the increased plasticity and compressibility of granulated GSF. In any case, the appearance of such a transient metastable polymorph emphasizes the complexity of the polymorph landscape and the importance of thorough crystal form screening.

## Supporting Information Available

Crystal data of the new and reported GSF solid phases and intermolecular and lattice energies of the new solid forms of GSF (PDF)
