# Supplementary material for: New Solid Forms of Griseofulvin: A Solvate and a Relict Polymorph Related to Reported Solvates
Source: Cryst Growth Des. 2023 Nov 15;23(12):8953–61. doi: 10.1021/acs.cgd.3c01011 (PMC10704485; doi:10.1021/acs.cgd.3c01011)
Supplement: Supplementary file 1 — cg3c01011_si_001.pdf [file cg3c01011_si_001.pdf]

New solid forms of griseofulvin: a solvate and a relict polymorph related to reported solvates

Mariana O. Diniz[1, 2], *Enrico Spoletti*[2], *Peuli Ghosh*[1, 2], *Matteo Lusi*[2], *Michael Svärd*[3], *Åke Rasmuson*[1,2,3], *Sarah P. Hudson*[1, 2]*

[1] SSPC, the Science Foundation Ireland Research Centre for Pharmaceuticals, University of Limerick, Limerick, V94 T9PX, Ireland.

[2] Department of Chemical Sciences, Bernal Institute, University of Limerick, Limerick, V94 T9PX, Ireland.

[3] Department of Chemical Engineering, KTH Royal Institute of Technology, Stockholm, SE-100 44, Sweden.

Supporting information

Table S1. Crystallographic parameters of polymorphic forms of GSF.

	Form VI		***Form I***	***Form II***	***Form III***	***Form IV***	***Form V***
**CSD code**	This work		GRISFL	GRISFL08	GRISFL16	GRISFL22	GRISFL36
**Year discovered**	This work		1947[1]	2013[2]	2013[2]	2022[3]	2022[3]
**Method**[a]	SC		SC	MC	MC	MC	MC
**Year that structure was solved**	This work		1977[4]	2018[5]	2020[6]	2022[3]	2022[3]
**T (K)**	150	298	298	298	298	298	298
**Crystal system**	monoclinic		tetragonal	orthorhombic	triclinic	orthorhombic	monoclinic
**Space group**	$P2_1$		$P4_1$	$P2_12_12_1$	$P1$	$P2_12_12_1$	$P2_1$
a **(Å)**	9.3824 (6)	9.4957 (4)	8.962(1)	11.799(8)	11.3540 (2)	8.46010 (1)	11.86930 (7)
b **(Å)**	8.6221 (5)	8.6772 (4)	8.962(1)	12.007(8)	11.9110 (2)	11.8293 (2)	8.43948 (4)
c **(Å)**	11.8304 (7)	11.8614 (5)	19.895(5)	24.359(16)	12.7608 (2)	51.6346 (15)	25.72097 (14)
α **(°)**	90	90	90	90	79.8440 (10)	90	90
β **(°)**	113.240 (3)	113.465 (2)	90	90	89.1950 (10)	90	90.1611 (6)
γ **(°)**	90	90	90	90	83.4730 (10)	90	90
**V (Å^3)**	879.38 (9)	896.51 (7)	1597.92	3450.95	1687.66	5167.99	2576.48
**Z**	2	2	4	8	4	12	6
**Z'**	1	1	1	2	4	3	3
**υ_0 (Å^3)**[c]	439.69	448.25	399.48	431.37	421.92	430.66	429.41
**d (g cm^{-3})**	1.332	1.307	1.463	1.367	1.388	1.358	1.363
**T_m (K)**	[b]		494	488	480	478	462

[a] Methods of polymorph discovery: SC, solution crystallisation; MC, melt crystallisation.

[b] Form VI transformed to Form I before melting.

[c] Molecular volume = cell volume (V) / formula units in unit cell (Z).

Table S2. Crystallographic parameters of solvated forms of GSF.

Solvate	***GSF-nBuAc*** *(this work)*	**GSF - benzene** [a]	***GSF - chloroform***	***GSF – acetonitrile***	***GSF – nitromethane***	***GSF – nitroethane (2:1)***	***GSF – nitroethane (1:1)***	***GSF – 1,1-dichloroethane***	***GSF – dichloromethane***	***GSF – bromochloromethane*** [a]	***GSF – dibromomethane*** [a]	***GSF – bromoethane*** [a]
**Chemical Formula**	$C_{17}H_{17}ClO_6$,0.5$C_6H_{12}O_2$	$C_{17}H_{17}ClO_6$,C_6H_6	$C_{17}H_{17}ClO_6$,$CHCl_3$	$C_{17}H_{17}ClO_6$,C_2H_3N	$C_{17}H_{17}ClO_6$,CH_3NO_2	$C_{17}H_{17}ClO_6$, 0.5$C_2H_5NO_2$	$C_{17}H_{17}ClO_6$,$C_2H_5NO_2$	$C_{17}H_{17}ClO_6$,$C_2H_4Cl_2$	$C_{17}H_{17}ClO_6$,CH_2Cl_2	$C_{17}H_{17}ClO_6$,CH_2BrCl	$C_{17}H_{17}ClO_6$,CH_2Br_2	$C_{17}H_{17}ClO_6$,C_2H_5Br
**MW (g mol^{-1})**	410.83	430.88	472.14	393.81	413.80	390.29	427.83	451.72	437.70	482.15	526.60	461.73
**CSD code**	This work	GRISBZ	MATZEO	PINMOQ	PINMUW	PINNAD	PINNEH	VADGOW	VADGUC01	VADHAJ	VADHEN	VADHIR
**Year**	This work	1979 [7]	1979[8]	2014[9]	2014[9]	2014[9]	2014[9]	1988[10]	2019[11]	1988[10]	1988[10]	1988[10]
**T (K)**	150	RT	RT	110	110	163	110	RT	100	RT	RT	RT
**Crystal system**	Triclinic	Monoclinic	Orthorhombic	Monoclinic	Orthorhombic	Orthorhombic	Monoclinic	Orthorhombic	Triclinic	Triclinic	Triclinic	Triclinic
**Space group**	$P1$	$P2_1$	$P2_12_12_1$	$P2_1$	$P2_12_12_1$	$P2_12_12_1$	$C2$	$P2_12_12_1$	$P1$	-	-	-
a **(Å)**	8.6087 (3)	13.28	8.671 (2)	11.783 (2)	8.5456 (17)	8.6852 (17)	21.931 (4)	11.8995 (1)	8.5004 (6)	11.754 (5)	11.825 (9)	11.819 (5)
b **(Å)**	11.7252 (4)	8.80	11.890 (2)	8.5387 (17)	11.740 (2)	15.047 (3)	8.6541 (17)	20.720 (3)	11.6660 (8)	11.908 (6)	12.015 (10)	12.009 (6)
c **(Å)**	11.7453 (4)	11.80	20.81 (4)	19.297 (4)	19.269 (4)	27.605 (6)	11.797 (2)	8.720 (12)	11.6705 (11)	8.648 (4)	8.679 (5)	8.693 (3)
α **(°)**	113.2290 (1)	90	90	90	90	90	90	90	111.795 (4)	111.18 (3)	111.28 (5)	111.19 (4)
β **(°)**	90.0600 (1)	100	90	96.03 (3)	90	90	117.57 (3)	90	110.833 (3)	89.99 (3)	89.69(5)	89.91 (4)
γ **(°)**	110.9930 (1)	90	90	90	90	90	90	90	94.170 (3)	66.41 (3)	65.44(5)	65.67 (4)
**V (Å^3)**	1003.04	1358.00	2145.47	1930.76	1933.20	3607.59	1984.74	2149.17	975.973	1019.40	1027.14	1031.67
**Z**	2	2	4	4	4	8	4	4	2	-	-	-
**Z'**	2	1	1	2	1	2	1	1	2	-	-	-
**υ_0 (Å^3)**[b]	501.52	679.00	536.37	482.69	483.30	450.95	496.18	537.29	487.99	-	-	-
**d (g cm^{-3})**	1.360	1.054	1.462	1.355	1.422	1.437	1.432	1.396	1.489	1.571	1.703	1.486

[a] No coordinates available. Full crystal structure was not determined.

[b] Molecular volume = cell volume (V) / formula units in unit cell (Z).

Table S3. Intermolecular interaction energies of GSF Form I calculated for a 3.8 Å cluster of molecules using B3LYP/6-31G(d,p). Both the total energy (E(tot)) and electrostatic (E(ele)), polarization (E(pol)), dispersion (E(dis)), and exchange repulsion (E(rep)) components of the energy (kJ/mol) are listed. R indicated the distance between centres of mass of the pair of molecules (Å), the total intermolecular interaction energy for a given molecule is the sum of the electrostatic, polarization, dispersion, and exchange-repulsion components with scale factors of 1.057, 0.740, 0.871, and 0.618, respectively.

	N	Symop	R	Electron Density	E_ele	E_pol	E_dis	E_rep	E_tot
	2	y, -x, z+3/4	7.01	B3LYP/6-31G(d,p)	-29.5	-11.9	-57.8	45.9	-62.0
	2	-y, x, z+1/4	6.77	B3LYP/6-31G(d,p)	-8.7	-2.2	-31.6	22.6	-24.4
	2	x, y, z	8.91	B3LYP/6-31G(d,p)	-2.5	-1.6	-19.3	7.4	-16.0
	2	x, y, z	8.91	B3LYP/6-31G(d,p)	-2.1	-2.3	-10.9	11.4	-6.4
	2	-x, -y, z+1/2	10.50	B3LYP/6-31G(d,p)	-10.7	-4.3	-18.7	12.6	-23.0
	2	y, -x, z+3/4	9.78	B3LYP/6-31G(d,p)	-11.4	-6.8	-23.2	16.9	-26.8
	2	-y, x, z+1/4	9.61	B3LYP/6-31G(d,p)	2.0	-2.7	-20.0	11.3	-10.3

Table S4. Intermolecular interaction energies of GSF Form VI calculated for a 3.8 Å cluster of molecules using B3LYP/6-31G(d,p). Both the total energy (E(tot)) and electrostatic (E(ele)), polarization (E(pol)), dispersion (E(dis)), and exchange repulsion (E(rep)) components of the energy (kJ/mol) are listed. R indicated the distance between centres of mass of the pair of molecules (Å), the total intermolecular interaction energy for a given molecule is the sum of the electrostatic, polarization, dispersion, and exchange-repulsion components with scale factors of 1.057, 0.740, 0.871, and 0.618, respectively.

	N	Symop	R	Electron Density	E_ele	E_pol	E_dis	E_rep	E_tot
	2	x, y, z	11.85	B3LYP/6-31G(d,p)	-20.8	-7.1	-16.0	17.9	-30.2
	2	-x, y+1/2, -z	8.05	B3LYP/6-31G(d,p)	-15.6	-4.5	-39.9	24.4	-39.5
	2	-x, y+1/2, -z	11.94	B3LYP/6-31G(d,p)	0.0	-0.6	-2.9	0.1	-2.9
	2	x, y, z	8.62	B3LYP/6-31G(d,p)	-2.5	-3.5	-15.0	17.1	-7.8
	2	-x, y+1/2, -z	7.24	B3LYP/6-31G(d,p)	-15.3	-3.1	-28.7	23.6	-28.9
	2	-x, y+1/2, -z	7.94	B3LYP/6-31G(d,p)	-12.3	-5.2	-32.8	22.8	-31.4
	2	x, y, z	9.38	B3LYP/6-31G(d,p)	0.9	-0.7	-9.9	4.4	-5.4

Table S5. Intermolecular interaction energies of GSF-nBuAc desolvated calculated for GSF molecule A at 3.8 Å cluster of molecules using B3LYP/6-31G(d,p). Both the total energy (E(tot)) and electrostatic (E(ele)), polarization (E(pol)), dispersion (E(dis)), and exchange repulsion (E(rep)) components of the energy (kJ/mol) are listed. R indicated the distance between centres of mass of the pair of molecules (Å), the total intermolecular interaction energy for a given molecule is the sum of the electrostatic, polarization, dispersion, and exchange-repulsion components with scale factors of 1.057, 0.740, 0.871, and 0.618, respectively.

	N	Symop	R	Electron Density	E_ele	E_pol	E_dis	E_rep	E_tot
	1	-	6.58	B3LYP/6-31G(d,p)	-6.8	-1.0	-35.7	19.2	-27.1
	1	-	12.12	B3LYP/6-31G(d,p)	-4.6	-2.2	-5.6	0.0	-11.4
	2	x, y, z	8.61	B3LYP/6-31G(d,p)	-1.9	-3.6	-15.3	17.2	-7.4
	1	-	7.93	B3LYP/6-31G(d,p)	-15.6	-5.0	-28.6	19.0	-33.4
	2	x, y, z	11.75	B3LYP/6-31G(d,p)	-19.5	-7.5	-15.6	17.3	-29.1
	1	-	8.24	B3LYP/6-31G(d,p)	-16.1	-4.5	-40.6	26.2	-39.6
	1	-	8.24	B3LYP/6-31G(d,p)	-15.6	-4.5	-40.0	25.1	-39.2
	1	-	7.92	B3LYP/6-31G(d,p)	-16.9	-5.0	-29.7	21.2	-34.3

Table S6. Intermolecular interaction energies of GSF-nBuAc desolvated calculated for GSF molecule B at 3.8 Å cluster of molecules using B3LYP/6-31G(d,p). Both the total energy (E(tot)) and electrostatic (E(ele)), polarization (E(pol)), dispersion (E(dis)), and exchange repulsion (E(rep)) components of the energy (kJ/mol) are listed. R indicated the distance between centres of mass of the pair of molecules (Å), the total intermolecular interaction energy for a given molecule is the sum of the electrostatic, polarization, dispersion, and exchange-repulsion components with scale factors of 1.057, 0.740, 0.871, and 0.618, respectively.

	N	Symop	R	Electron Density	E_ele	E_pol	E_dis	E_rep	E_tot
	1	-	6.58	B3LYP/6-31G(d,p)	-6.8	-1.0	-35.7	19.2	-27.1
	1	-	12.12	B3LYP/6-31G(d,p)	-4.6	-2.2	-5.6	0.0	-11.4
	0	x, y, z	8.61	B3LYP/6-31G(d,p)	-1.9	-3.6	-15.3	17.2	-7.4

	1	-	7.93	B3LYP/6-31G(d,p)	-15.6	-5.0	-28.6	19.0	-33.4
	0	x, y, z	11.75	B3LYP/6-31G(d,p)	-19.5	-7.5	-15.6	17.3	-29.1
	1	-	8.24	B3LYP/6-31G(d,p)	-16.1	-4.5	-40.6	26.2	-39.6
	1	-	8.24	B3LYP/6-31G(d,p)	-15.6	-4.5	-40.0	25.1	-39.2
	1	-	7.92	B3LYP/6-31G(d,p)	-16.9	-5.0	-29.7	21.2	-34.3
	2	x, y, z	11.75	B3LYP/6-31G(d,p)	-17.4	-6.4	-16.1	17.9	-26.2
	2	x, y, z	8.61	B3LYP/6-31G(d,p)	-2.0	-3.8	-15.2	17.1	-7.5

Table S7. Lattice energy of GSF Form I calculated for a 20 Å cluster of molecules using B3LYP/6-31G(d,p) wavefunction; the total intermolecular interaction energy for a given molecule is the sum of the electrostatic, polarization, dispersion, and exchange-repulsion components with scale factors of 1.057, 0.740, 0.871, and 0.618, respectively.

	N	Symop	R	Electron Density	E_ele	E_pol	E_dis	E_rep	E_tot
	2	y, -x, z+3/4	25.40	B3LYP/6-31G(d,p)	0.2	-0.0	-0.0	0.0	0.2
	2	-x, -y, z+1/2	23.66	B3LYP/6-31G(d,p)	0.0	-0.0	-0.0	0.0	0.0
	2	-y, x, z+1/4	24.19	B3LYP/6-31G(d,p)	0.1	-0.0	-0.0	0.0	0.1
	2	x, y, z	25.21	B3LYP/6-31G(d,p)	0.2	-0.0	-0.0	0.0	0.2
	2	y, -x, z+3/4	18.00	B3LYP/6-31G(d,p)	0.4	-0.0	-0.1	0.0	0.3
	2	-y, x, z+1/4	24.95	B3LYP/6-31G(d,p)	-0.1	-0.0	-0.0	0.0	-0.1
	2	-y, x, z+1/4	27.90	B3LYP/6-31G(d,p)	-0.0	-0.0	-0.0	0.0	-0.1
	2	-x, -y, z+1/2	26.94	B3LYP/6-31G(d,p)	0.0	-0.0	-0.0	0.0	0.0
	2	-x, -y, z+1/2	19.27	B3LYP/6-31G(d,p)	0.0	-0.0	-0.0	0.0	0.0
	2	y, -x, z+3/4	23.08	B3LYP/6-31G(d,p)	0.5	-0.0	-0.0	0.0	0.5
	2	x, y, z	28.19	B3LYP/6-31G(d,p)	0.1	-0.0	-0.0	0.0	0.1
	2	x, y, z	19.93	B3LYP/6-31G(d,p)	0.4	-0.0	-0.0	0.0	0.4
	2	-y, x, z+1/4	20.73	B3LYP/6-31G(d,p)	0.1	-0.0	-0.0	0.0	0.0

	2	y, -x, z+3/4	14.54	B3LYP/6-31G(d,p)	2.5	-0.1	-0.4	0.0	2.3
	2	y, -x, z+3/4	20.11	B3LYP/6-31G(d,p)	-0.1	-0.0	-0.0	0.0	-0.1
	2	y, -x, z+3/4	28.95	B3LYP/6-31G(d,p)	-0.0	-0.0	-0.0	0.0	-0.0
	2	-y, x, z+1/4	25.02	B3LYP/6-31G(d,p)	-0.1	-0.0	-0.0	0.0	-0.1
	2	-x, -y, z+1/2	26.38	B3LYP/6-31G(d,p)	0.0	-0.0	-0.0	0.0	0.0
	2	-x, -y, z+1/2	18.49	B3LYP/6-31G(d,p)	-0.2	-0.0	-0.1	0.0	-0.3
	2	x, y, z	26.53	B3LYP/6-31G(d,p)	-0.4	-0.0	-0.0	0.0	-0.5
	2	y, -x, z+3/4	24.07	B3LYP/6-31G(d,p)	0.4	-0.0	-0.0	0.0	0.4
	2	x, y, z	26.74	B3LYP/6-31G(d,p)	0.1	-0.0	-0.0	0.0	0.1
	2	x, y, z	17.83	B3LYP/6-31G(d,p)	0.4	-0.0	-0.1	0.0	0.3
	2	-y, x, z+1/4	20.81	B3LYP/6-31G(d,p)	0.2	-0.0	-0.0	0.0	0.2
	2	y, -x, z+3/4	16.06	B3LYP/6-31G(d,p)	1.0	-0.1	-0.2	0.0	0.8
	2	y, -x, z+3/4	26.94	B3LYP/6-31G(d,p)	0.1	-0.0	-0.0	0.0	0.1
	2	-x, -y, z+1/2	21.71	B3LYP/6-31G(d,p)	0.3	-0.0	-0.0	0.0	0.3
	2	-y, x, z+1/4	28.08	B3LYP/6-31G(d,p)	-0.1	-0.0	-0.0	0.0	-0.1
	2	y, -x, z+3/4	28.01	B3LYP/6-31G(d,p)	0.1	-0.0	-0.0	0.0	0.1
	2	x, y, z	19.93	B3LYP/6-31G(d,p)	0.1	-0.0	-0.0	0.0	0.0
	2	x, y, z	28.19	B3LYP/6-31G(d,p)	0.0	-0.0	-0.0	0.0	0.0
	2	-y, x, z+1/4	24.40	B3LYP/6-31G(d,p)	0.2	-0.0	-0.0	0.0	0.2
	2	y, -x, z+3/4	21.53	B3LYP/6-31G(d,p)	-0.3	-0.0	-0.0	0.0	-0.3
	2	-y, x, z+1/4	22.51	B3LYP/6-31G(d,p)	-0.1	-0.0	-0.0	0.0	-0.1
	2	-y, x, z+1/4	28.66	B3LYP/6-31G(d,p)	-0.0	-0.0	-0.0	0.0	-0.0
	2	-x, -y, z+1/2	19.10	B3LYP/6-31G(d,p)	0.1	-0.0	-0.0	0.0	0.0
	2	x, y, z	19.93	B3LYP/6-31G(d,p)	0.2	-0.0	-0.0	0.0	0.1

	2	-y, x, z+1/4	17.71	B3LYP/6-31G(d,p)	0.2	-0.0	-0.0	0.0	0.2
	2	-y, x, z+1/4	25.06	B3LYP/6-31G(d,p)	0.1	-0.0	-0.0	0.0	0.1
	2	y, -x, z+3/4	12.71	B3LYP/6-31G(d,p)	0.5	-0.0	-0.5	0.0	0.1
	2	y, -x, z+3/4	22.74	B3LYP/6-31G(d,p)	-0.2	-0.0	-0.0	0.0	-0.2
	2	y, -x, z+3/4	18.83	B3LYP/6-31G(d,p)	-0.4	-0.0	-0.0	0.0	-0.5
	2	x, y, z	27.99	B3LYP/6-31G(d,p)	-0.1	-0.0	-0.0	0.0	-0.2
	2	y, -x, z+3/4	28.12	B3LYP/6-31G(d,p)	-0.1	-0.0	-0.0	0.0	-0.1
	2	x, y, z	23.35	B3LYP/6-31G(d,p)	-0.2	-0.0	-0.0	0.0	-0.2
	2	-y, x, z+1/4	18.74	B3LYP/6-31G(d,p)	-0.7	-0.0	-0.1	0.0	-0.8
	2	-x, -y, z+1/2	13.27	B3LYP/6-31G(d,p)	0.9	-0.0	-0.4	0.0	0.6
	2	x, y, z	12.61	B3LYP/6-31G(d,p)	-0.0	-0.1	-0.6	0.0	-0.7
	2	-y, x, z+1/4	12.58	B3LYP/6-31G(d,p)	-0.1	-0.0	-0.5	0.0	-0.5
	2	y, -x, z+3/4	7.01	B3LYP/6-31G(d,p)	-29.5	-11.9	-57.8	45.9	-62.0
	2	x, y, z	23.35	B3LYP/6-31G(d,p)	0.1	-0.0	-0.0	0.0	0.1
	2	y, -x, z+3/4	15.56	B3LYP/6-31G(d,p)	-1.4	-0.1	-0.2	0.0	-1.6
	2	y, -x, z+3/4	28.94	B3LYP/6-31G(d,p)	-0.3	-0.0	-0.0	0.0	-0.3
	2	x, y, z	21.58	B3LYP/6-31G(d,p)	-0.8	-0.0	-0.0	0.0	-0.9
	2	-x, -y, z+1/2	12.11	B3LYP/6-31G(d,p)	-0.3	-0.4	-1.1	0.0	-1.5
	2	x, y, z	8.91	B3LYP/6-31G(d,p)	-2.5	-1.6	-19.3	7.4	-16.0
	2	y, -x, z+3/4	9.78	B3LYP/6-31G(d,p)	-11.4	-6.8	-23.2	16.9	-26.8
	2	y, -x, z+3/4	21.24	B3LYP/6-31G(d,p)	0.2	-0.0	-0.0	0.0	0.2
	2	x, y, z	21.58	B3LYP/6-31G(d,p)	0.2	-0.0	-0.0	0.0	0.2
	2	x, y, z	26.53	B3LYP/6-31G(d,p)	0.1	-0.0	-0.0	0.0	0.2
	2	y, -x, z+3/4	16.99	B3LYP/6-31G(d,p)	-0.1	-0.0	-0.1	0.0	-0.2

	2	x, y, z	27.99	B3LYP/6-31G(d,p)	-0.4	-0.0	-0.0	0.0	-0.5
	2	x, y, z	23.35	B3LYP/6-31G(d,p)	-0.7	-0.0	-0.0	0.0	-0.8
	2	-x, -y, z+1/2	16.61	B3LYP/6-31G(d,p)	0.0	-0.1	-0.1	0.0	-0.1
	2	x, y, z	12.61	B3LYP/6-31G(d,p)	1.5	-0.2	-0.6	0.0	0.9
	2	x, y, z	25.21	B3LYP/6-31G(d,p)	0.1	-0.0	-0.0	0.0	0.1
	2	y, -x, z+3/4	17.35	B3LYP/6-31G(d,p)	-1.0	-0.0	-0.1	0.0	-1.1
	2	x, y, z	23.35	B3LYP/6-31G(d,p)	0.2	-0.0	-0.0	0.0	0.2
	2	y, -x, z+3/4	22.23	B3LYP/6-31G(d,p)	0.2	-0.0	-0.0	0.0	0.2
	2	-x, -y, z+1/2	23.75	B3LYP/6-31G(d,p)	0.2	-0.0	-0.0	0.0	0.2
	2	x, y, z	19.93	B3LYP/6-31G(d,p)	0.4	-0.0	-0.0	0.0	0.4
	2	y, -x, z+3/4	25.62	B3LYP/6-31G(d,p)	0.2	-0.0	-0.0	0.0	0.2
	2	-y, x, z+1/4	22.71	B3LYP/6-31G(d,p)	0.2	-0.0	-0.0	0.0	0.2
	2	-x, -y, z+1/2	26.00	B3LYP/6-31G(d,p)	0.0	-0.0	-0.0	0.0	0.0
	2	x, y, z	27.99	B3LYP/6-31G(d,p)	0.0	-0.0	-0.0	0.0	-0.0
	2	-y, x, z+1/4	19.86	B3LYP/6-31G(d,p)	-0.3	-0.0	-0.0	0.0	-0.4
	2	-y, x, z+1/4	26.63	B3LYP/6-31G(d,p)	-0.1	-0.0	-0.0	0.0	-0.1
	2	-x, -y, z+1/2	18.12	B3LYP/6-31G(d,p)	0.0	-0.0	-0.0	0.0	-0.0
	2	x, y, z	17.83	B3LYP/6-31G(d,p)	0.4	-0.0	-0.0	0.0	0.4
	2	-y, x, z+1/4	14.19	B3LYP/6-31G(d,p)	0.6	-0.0	-0.2	0.0	0.5
	2	x, y, z	26.53	B3LYP/6-31G(d,p)	-0.1	-0.0	-0.0	0.0	-0.1
	2	y, -x, z+3/4	25.07	B3LYP/6-31G(d,p)	-0.2	-0.0	-0.0	0.0	-0.2
	2	x, y, z	21.58	B3LYP/6-31G(d,p)	0.0	-0.0	-0.0	0.0	0.0
	2	-y, x, z+1/4	15.46	B3LYP/6-31G(d,p)	-1.5	-0.1	-0.3	0.0	-1.9
	2	-x, -y, z+1/2	11.82	B3LYP/6-31G(d,p)	-0.4	-0.1	-1.5	0.0	-1.8

	2	y, -x, z+3/4	25.00	B3LYP/6-31G(d,p)	-0.1	-0.0	-0.0	0.0	-0.1
	2	x, y, z	8.91	B3LYP/6-31G(d,p)	-2.1	-2.3	-10.9	11.4	-6.4
	2	-y, x, z+1/4	6.77	B3LYP/6-31G(d,p)	-8.7	-2.2	-31.6	22.6	-24.4
	2	x, y, z	21.58	B3LYP/6-31G(d,p)	-0.2	-0.0	-0.0	0.0	-0.3
	2	y, -x, z+3/4	25.98	B3LYP/6-31G(d,p)	-0.4	-0.0	-0.0	0.0	-0.5
	2	x, y, z	19.65	B3LYP/6-31G(d,p)	-0.2	-0.0	-0.0	0.0	-0.3
	2	-x, -y, z+1/2	10.50	B3LYP/6-31G(d,p)	-10.7	-4.3	-18.7	12.6	-23.0
	2	y, -x, z+3/4	25.92	B3LYP/6-31G(d,p)	-0.2	-0.0	-0.0	0.0	-0.2
	2	y, -x, z+3/4	9.61	B3LYP/6-31G(d,p)	2.0	-2.7	-20.0	11.3	-10.3
	2	y, -x, z+3/4	16.89	B3LYP/6-31G(d,p)	-0.1	-0.0	-0.1	0.0	-0.3
	2	y, -x, z+3/4	29.67	B3LYP/6-31G(d,p)	-0.4	-0.0	-0.0	0.0	-0.4
	2	-x, -y, z+1/2	15.48	B3LYP/6-31G(d,p)	-2.3	-0.1	-0.2	0.0	-2.7
	2	x, y, z	27.99	B3LYP/6-31G(d,p)	-0.2	-0.0	-0.0	0.0	-0.2
	2	y, -x, z+3/4	17.25	B3LYP/6-31G(d,p)	0.6	-0.0	-0.1	0.0	0.5
	2	y, -x, z+3/4	22.15	B3LYP/6-31G(d,p)	0.2	-0.0	-0.0	0.0	0.2
	2	-x, -y, z+1/2	22.98	B3LYP/6-31G(d,p)	-0.2	-0.0	-0.0	0.0	-0.2
	2	y, -x, z+3/4	25.80	B3LYP/6-31G(d,p)	-0.1	-0.0	-0.0	0.0	-0.1
	2	x, y, z	26.53	B3LYP/6-31G(d,p)	0.1	-0.0	-0.0	0.0	0.1
	2	x, y, z	26.74	B3LYP/6-31G(d,p)	0.1	-0.0	-0.0	0.0	0.1
	2	-y, x, z+1/4	23.71	B3LYP/6-31G(d,p)	0.2	-0.0	-0.0	0.0	0.2
	2	-y, x, z+1/4	21.00	B3LYP/6-31G(d,p)	-0.1	-0.0	-0.0	0.0	-0.1
	2	-y, x, z+1/4	27.48	B3LYP/6-31G(d,p)	-0.0	-0.0	-0.0	0.0	-0.0
	2	-x, -y, z+1/2	21.23	B3LYP/6-31G(d,p)	0.0	-0.0	-0.0	0.0	-0.0
	2	-y, x, z+1/4	15.74	B3LYP/6-31G(d,p)	0.9	-0.0	-0.1	0.0	0.9

	2	-y, x, z+1/4	28.76	B3LYP/6-31G(d,p)	-0.0	-0.0	-0.0	0.0	-0.0
	2	-x, -y, z+1/2	16.20	B3LYP/6-31G(d,p)	0.1	-0.0	-0.2	0.0	-0.1
	2	-x, -y, z+1/2	15.26	B3LYP/6-31G(d,p)	0.5	-0.1	-0.2	0.0	0.2
	2	y, -x, z+3/4	29.61	B3LYP/6-31G(d,p)	-0.2	-0.0	-0.0	0.0	-0.2
	2	-x, -y, z+1/2	19.03	B3LYP/6-31G(d,p)	-0.1	-0.0	-0.0	0.0	-0.1
	2	y, -x, z+3/4	21.29	B3LYP/6-31G(d,p)	0.5	-0.0	-0.0	0.0	0.5
	2	y, -x, z+3/4	25.42	B3LYP/6-31G(d,p)	0.1	-0.0	-0.0	0.0	0.1
	2	-x, -y, z+1/2	25.50	B3LYP/6-31G(d,p)	-0.0	-0.0	-0.0	0.0	-0.1
	2	y, -x, z+3/4	25.73	B3LYP/6-31G(d,p)	0.1	-0.0	-0.0	0.0	0.1
	2	-y, x, z+1/4	27.71	B3LYP/6-31G(d,p)	0.2	-0.0	-0.0	0.0	0.2
	2	-x, -y, z+1/2	27.06	B3LYP/6-31G(d,p)	0.1	-0.0	-0.0	0.0	0.1
	2	-x, -y, z+1/2	23.32	B3LYP/6-31G(d,p)	0.2	-0.0	-0.0	0.0	0.2
	2	-x, -y, z+1/2	22.68	B3LYP/6-31G(d,p)	0.3	-0.0	-0.0	0.0	0.3
	2	-x, -y, z+1/2	25.37	B3LYP/6-31G(d,p)	0.2	-0.0	-0.0	0.0	0.2
	2	y, -x, z+3/4	28.59	B3LYP/6-31G(d,p)	0.2	-0.0	-0.0	0.0	0.2

Table S8. Lattice energy of GSF Form VI calculated for a 20 Å cluster of molecules using B3LYP/6-31G(d,p) wavefunction; the total intermolecular interaction energy for a given molecule is the sum of the electrostatic, polarization, dispersion, and exchange-repulsion components with scale factors of 1.057, 0.740, 0.871, and 0.618, respectively.

	N	Symop	R	Electron Density	E_ele	E_pol	E_dis	E_rep	E_tot
	2	x, y, z	27.27	B3LYP/6-31G(d,p)	0.0	-0.0	-0.0	0.0	0.0
	2	-x, y+1/2, -z	29.25	B3LYP/6-31G(d,p)	0.2	-0.0	-0.0	0.0	0.2
	2	x, y, z	28.75	B3LYP/6-31G(d,p)	-0.3	-0.0	-0.0	0.0	-0.3
	2	x, y, z	25.87	B3LYP/6-31G(d,p)	0.1	-0.0	-0.0	0.0	0.1
	2	-x, y+1/2, -z	19.91	B3LYP/6-31G(d,p)	-0.0	-0.0	-0.0	0.0	-0.1

	2	-x, y+1/2, -z	25.73	B3LYP/6-31G(d,p)	0.2	-0.0	-0.0	0.0	0.2
	2	x, y, z	29.31	B3LYP/6-31G(d,p)	-0.2	-0.0	-0.0	0.0	-0.2
	2	-x, y+1/2, -z	22.27	B3LYP/6-31G(d,p)	0.2	-0.0	-0.0	0.0	0.2
	2	-x, y+1/2, -z	30.97	B3LYP/6-31G(d,p)	-0.0	-0.0	-0.0	0.0	-0.0
	2	x, y, z	24.78	B3LYP/6-31G(d,p)	0.0	-0.0	-0.0	0.0	0.0
	2	-x, y+1/2, -z	23.34	B3LYP/6-31G(d,p)	-0.2	-0.0	-0.0	0.0	-0.2
	2	-x, y+1/2, -z	17.06	B3LYP/6-31G(d,p)	-0.9	-0.0	-0.1	0.0	-1.0
	2	-x, y+1/2, -z	22.66	B3LYP/6-31G(d,p)	0.8	-0.0	-0.0	0.0	0.9
	2	x, y, z	25.22	B3LYP/6-31G(d,p)	-0.6	-0.0	-0.0	0.0	-0.6
	2	x, y, z	19.78	B3LYP/6-31G(d,p)	-0.1	-0.0	-0.0	0.0	-0.1
	2	x, y, z	30.01	B3LYP/6-31G(d,p)	-0.2	-0.0	-0.0	0.0	-0.2
	2	-x, y+1/2, -z	30.17	B3LYP/6-31G(d,p)	0.4	-0.0	-0.0	0.0	0.4
	2	-x, y+1/2, -z	18.63	B3LYP/6-31G(d,p)	1.7	-0.1	-0.1	0.0	1.7
	2	x, y, z	20.65	B3LYP/6-31G(d,p)	0.3	-0.0	-0.0	0.0	0.3
	2	-x, y+1/2, -z	11.94	B3LYP/6-31G(d,p)	0.0	-0.6	-2.9	0.1	-2.9
	2	-x, y+1/2, -z	19.14	B3LYP/6-31G(d,p)	-0.5	-0.0	-0.0	0.0	-0.6
	2	-x, y+1/2, -z	14.75	B3LYP/6-31G(d,p)	-0.8	-0.0	-0.1	0.0	-1.0
	2	-x, y+1/2, -z	23.64	B3LYP/6-31G(d,p)	-0.3	-0.0	-0.0	0.0	-0.3
	2	x, y, z	23.70	B3LYP/6-31G(d,p)	-0.9	-0.0	-0.0	0.0	-0.9
	2	x, y, z	17.80	B3LYP/6-31G(d,p)	0.1	-0.0	-0.1	0.0	-0.0
	2	x, y, z	18.76	B3LYP/6-31G(d,p)	0.5	-0.0	-0.0	0.0	0.5
	2	-x, y+1/2, -z	23.93	B3LYP/6-31G(d,p)	-0.2	-0.0	-0.0	0.0	-0.2
	2	x, y, z	25.22	B3LYP/6-31G(d,p)	-0.4	-0.0	-0.0	0.0	-0.5
	2	x, y, z	19.78	B3LYP/6-31G(d,p)	-0.0	-0.0	-0.0	0.0	-0.1

	2	x, y, z	20.65	B3LYP/6-31G(d,p)	0.3	-0.0	-0.0	0.0	0.3
	2	x, y, z	27.27	B3LYP/6-31G(d,p)	0.1	-0.0	-0.0	0.0	0.1
	2	x, y, z	24.78	B3LYP/6-31G(d,p)	0.0	-0.0	-0.0	0.0	0.0
	2	x, y, z	25.48	B3LYP/6-31G(d,p)	0.2	-0.0	-0.0	0.0	0.2
	2	-x, y+1/2, -z	22.60	B3LYP/6-31G(d,p)	-0.3	-0.0	-0.0	0.0	-0.4
	2	x, y, z	20.92	B3LYP/6-31G(d,p)	0.2	-0.0	-0.0	0.0	0.1
	2	-x, y+1/2, -z	28.16	B3LYP/6-31G(d,p)	-0.1	-0.0	-0.0	0.0	-0.1
	2	x, y, z	27.75	B3LYP/6-31G(d,p)	-0.1	-0.0	-0.0	0.0	-0.1
	2	-x, y+1/2, -z	22.49	B3LYP/6-31G(d,p)	-0.0	-0.0	-0.0	0.0	-0.0
	2	x, y, z	19.63	B3LYP/6-31G(d,p)	0.4	-0.0	-0.0	0.0	0.4
	2	-x, y+1/2, -z	24.26	B3LYP/6-31G(d,p)	-0.3	-0.0	-0.0	0.0	-0.3
	2	-x, y+1/2, -z	14.61	B3LYP/6-31G(d,p)	-1.5	-0.0	-0.3	0.0	-1.9
	2	-x, y+1/2, -z	22.32	B3LYP/6-31G(d,p)	-0.2	-0.0	-0.0	0.0	-0.3
	2	x, y, z	25.48	B3LYP/6-31G(d,p)	0.2	-0.0	-0.0	0.0	0.2
	2	-x, y+1/2, -z	14.18	B3LYP/6-31G(d,p)	-0.5	-0.0	-0.2	0.0	-0.8
	2	x, y, z	24.76	B3LYP/6-31G(d,p)	0.2	-0.0	-0.0	0.0	0.2
	2	-x, y+1/2, -z	21.65	B3LYP/6-31G(d,p)	-0.0	-0.0	-0.0	0.0	-0.0
	2	x, y, z	23.39	B3LYP/6-31G(d,p)	-0.5	-0.0	-0.0	0.0	-0.5
	2	x, y, z	14.65	B3LYP/6-31G(d,p)	-0.8	-0.3	-0.8	0.0	-1.8
	2	-x, y+1/2, -z	28.84	B3LYP/6-31G(d,p)	0.3	-0.0	-0.0	0.0	0.3
	2	-x, y+1/2, -z	18.90	B3LYP/6-31G(d,p)	0.3	-0.0	-0.0	0.0	0.3
	2	x, y, z	12.74	B3LYP/6-31G(d,p)	0.9	-0.0	-0.5	0.0	0.5
	2	-x, y+1/2, -z	8.05	B3LYP/6-31G(d,p)	-15.6	-4.5	-39.9	24.4	-39.5
	2	x, y, z	19.75	B3LYP/6-31G(d,p)	0.3	-0.0	-0.0	0.0	0.3

	2	-x, y+1/2, -z	7.24	B3LYP/6-31G(d,p)	-15.3	-3.1	-28.7	23.6	-28.9
	2	x, y, z	27.23	B3LYP/6-31G(d,p)	0.2	-0.0	-0.0	0.0	0.2
	2	-x, y+1/2, -z	17.89	B3LYP/6-31G(d,p)	-0.0	-0.0	-0.1	0.0	-0.1
	2	x, y, z	21.74	B3LYP/6-31G(d,p)	-0.8	-0.0	-0.0	0.0	-0.9
	2	x, y, z	11.85	B3LYP/6-31G(d,p)	-20.8	-7.1	-16.0	17.9	-30.2
	2	x, y, z	9.38	B3LYP/6-31G(d,p)	0.9	-0.7	-9.9	4.4	-5.4
	2	x, y, z	17.76	B3LYP/6-31G(d,p)	0.4	-0.0	-0.0	0.0	0.4
	2	x, y, z	25.83	B3LYP/6-31G(d,p)	0.2	-0.0	-0.0	0.0	0.2
	2	x, y, z	23.39	B3LYP/6-31G(d,p)	-0.4	-0.0	-0.0	0.0	-0.4
	2	x, y, z	14.65	B3LYP/6-31G(d,p)	-1.1	-0.1	-0.3	0.0	-1.5
	2	x, y, z	12.74	B3LYP/6-31G(d,p)	0.4	-0.1	-0.4	0.0	0.0
	2	x, y, z	19.75	B3LYP/6-31G(d,p)	0.3	-0.0	-0.0	0.0	0.3
	2	x, y, z	20.92	B3LYP/6-31G(d,p)	0.0	-0.0	-0.0	0.0	0.0
	2	x, y, z	27.75	B3LYP/6-31G(d,p)	-0.1	-0.0	-0.0	0.0	-0.1
	2	x, y, z	19.63	B3LYP/6-31G(d,p)	0.3	-0.0	-0.0	0.0	0.3
	2	x, y, z	24.76	B3LYP/6-31G(d,p)	0.2	-0.0	-0.0	0.0	0.2
	2	-x, y+1/2, -z	22.56	B3LYP/6-31G(d,p)	-0.2	-0.0	-0.0	0.0	-0.2
	2	x, y, z	25.87	B3LYP/6-31G(d,p)	0.2	-0.0	-0.0	0.0	0.2
	2	x, y, z	20.91	B3LYP/6-31G(d,p)	0.3	-0.0	-0.0	0.0	0.3
	2	-x, y+1/2, -z	28.34	B3LYP/6-31G(d,p)	-0.1	-0.0	-0.0	0.0	-0.1
	2	x, y, z	17.24	B3LYP/6-31G(d,p)	0.6	-0.0	-0.1	0.0	0.5
	2	-x, y+1/2, -z	24.68	B3LYP/6-31G(d,p)	-0.2	-0.0	-0.0	0.0	-0.2
	2	-x, y+1/2, -z	17.66	B3LYP/6-31G(d,p)	-0.4	-0.0	-0.0	0.0	-0.5
	2	x, y, z	20.91	B3LYP/6-31G(d,p)	0.2	-0.0	-0.0	0.0	0.2

	2	-x, y+1/2, -z	14.55	B3LYP/6-31G(d,p)	-0.2	-0.0	-0.2	0.0	-0.4
	2	-x, y+1/2, -z	19.79	B3LYP/6-31G(d,p)	0.4	-0.0	-0.0	0.0	0.4
	2	x, y, z	14.64	B3LYP/6-31G(d,p)	0.7	-0.1	-0.3	0.0	0.5
	2	x, y, z	25.18	B3LYP/6-31G(d,p)	-0.1	-0.0	-0.0	0.0	-0.1
	2	-x, y+1/2, -z	26.30	B3LYP/6-31G(d,p)	-0.0	-0.0	-0.0	0.0	-0.1
	2	-x, y+1/2, -z	23.30	B3LYP/6-31G(d,p)	0.0	-0.0	-0.0	0.0	0.0
	2	x, y, z	8.62	B3LYP/6-31G(d,p)	-2.5	-3.5	-15.0	17.1	-7.8
	2	-x, y+1/2, -z	12.77	B3LYP/6-31G(d,p)	0.2	-0.0	-0.3	0.0	-0.1
	2	x, y, z	14.64	B3LYP/6-31G(d,p)	0.6	-0.0	-0.2	0.0	0.4
	2	-x, y+1/2, -z	7.94	B3LYP/6-31G(d,p)	-12.3	-5.2	-32.8	22.8	-31.4
	2	x, y, z	25.18	B3LYP/6-31G(d,p)	-0.0	-0.0	-0.0	0.0	-0.1
	2	-x, y+1/2, -z	15.59	B3LYP/6-31G(d,p)	2.6	-0.2	-0.3	0.0	2.4
	2	x, y, z	23.66	B3LYP/6-31G(d,p)	-0.1	-0.0	-0.0	0.0	-0.1
	2	x, y, z	11.83	B3LYP/6-31G(d,p)	0.2	-0.2	-1.2	0.0	-1.0
	2	-x, y+1/2, -z	26.51	B3LYP/6-31G(d,p)	0.3	-0.0	-0.0	0.0	0.4
	2	-x, y+1/2, -z	24.21	B3LYP/6-31G(d,p)	-0.2	-0.0	-0.0	0.0	-0.2
	2	-x, y+1/2, -z	19.96	B3LYP/6-31G(d,p)	-0.3	-0.0	-0.0	0.0	-0.3
	2	-x, y+1/2, -z	22.16	B3LYP/6-31G(d,p)	0.2	-0.0	-0.0	0.0	0.2
	2	-x, y+1/2, -z	20.92	B3LYP/6-31G(d,p)	-0.1	-0.0	-0.0	0.0	-0.2
	2	-x, y+1/2, -z	15.81	B3LYP/6-31G(d,p)	-0.4	-0.0	-0.2	0.0	-0.6
	2	-x, y+1/2, -z	18.50	B3LYP/6-31G(d,p)	0.7	-0.0	-0.1	0.0	0.6
	2	-x, y+1/2, -z	26.73	B3LYP/6-31G(d,p)	0.5	-0.0	-0.0	0.0	0.6
	2	-x, y+1/2, -z	27.59	B3LYP/6-31G(d,p)	-0.1	-0.0	-0.0	0.0	-0.1
	2	-x, y+1/2, -z	24.75	B3LYP/6-31G(d,p)	-0.2	-0.0	-0.0	0.0	-0.2

	2	-x, y+1/2, -z	27.68	B3LYP/6-31G(d,p)	-0.0	-0.0	-0.0	0.0	-0.0
	2	-x, y+1/2, -z	24.85	B3LYP/6-31G(d,p)	-0.0	-0.0	-0.0	0.0	-0.1
	2	-x, y+1/2, -z	30.04	B3LYP/6-31G(d,p)	0.3	-0.0	-0.0	0.0	0.3

Table S9. Lattice energy of GSF-nBuAc desolvated calculated for GSF molecule A at 20 Å cluster of molecules using B3LYP/6-31G(d,p) wavefunction; the total intermolecular interaction energy for a given molecule is the sum of the electrostatic, polarization, dispersion, and exchange-repulsion components with scale factors of 1.057, 0.740, 0.871, and 0.618, respectively.

	N	Symop	R	Electron Density	E_ele	E_pol	E_dis	E_rep	E_tot
	1	-	22.63	B3LYP/6-31G(d,p)	-0.3	-0.0	-0.0	0.0	-0.4
	1	-	21.19	B3LYP/6-31G(d,p)	-0.3	-0.0	-0.0	0.0	-0.3
	1	-	25.73	B3LYP/6-31G(d,p)	0.1	-0.0	-0.0	0.0	0.1
	2	x, y, z	24.82	B3LYP/6-31G(d,p)	0.2	-0.0	-0.0	0.0	0.2
	1	-	22.70	B3LYP/6-31G(d,p)	-0.3	-0.0	-0.0	0.0	-0.3
	1	-	18.53	B3LYP/6-31G(d,p)	-0.5	-0.0	-0.0	0.0	-0.5
	2	x, y, z	17.84	B3LYP/6-31G(d,p)	0.4	-0.0	-0.0	0.0	0.4
	2	x, y, z	24.24	B3LYP/6-31G(d,p)	0.3	-0.0	-0.0	0.0	0.3
	1	-	26.75	B3LYP/6-31G(d,p)	-0.2	-0.0	-0.0	0.0	-0.2
	1	-	21.15	B3LYP/6-31G(d,p)	0.1	-0.0	-0.0	0.0	0.1
	2	x, y, z	17.01	B3LYP/6-31G(d,p)	0.5	-0.0	-0.1	0.0	0.5
	2	x, y, z	28.89	B3LYP/6-31G(d,p)	-0.0	-0.0	-0.0	0.0	-0.0
	2	x, y, z	23.16	B3LYP/6-31G(d,p)	-0.3	-0.0	-0.0	0.0	-0.3
	1	-	22.64	B3LYP/6-31G(d,p)	-0.3	-0.0	-0.0	0.0	-0.4
	2	x, y, z	25.83	B3LYP/6-31G(d,p)	0.3	-0.0	-0.0	0.0	0.3
	1	-	22.54	B3LYP/6-31G(d,p)	-0.1	-0.0	-0.0	0.0	-0.1

	2	x, y, z	17.22	B3LYP/6-31G(d,p)	0.9	-0.0	-0.1	0.0	0.8
	2	x, y, z	20.85	B3LYP/6-31G(d,p)	0.2	-0.0	-0.0	0.0	0.2
	2	x, y, z	25.95	B3LYP/6-31G(d,p)	0.2	-0.0	-0.0	0.0	0.2
	1	-	18.99	B3LYP/6-31G(d,p)	-0.4	-0.0	-0.0	0.0	-0.5
	1	-	26.46	B3LYP/6-31G(d,p)	0.0	-0.0	-0.0	0.0	0.0
	2	x, y, z	21.85	B3LYP/6-31G(d,p)	0.3	-0.0	-0.0	0.0	0.3
	1	-	17.24	B3LYP/6-31G(d,p)	-0.5	-0.0	-0.1	0.0	-0.6
	2	x, y, z	20.00	B3LYP/6-31G(d,p)	0.4	-0.0	-0.0	0.0	0.4
	2	x, y, z	23.60	B3LYP/6-31G(d,p)	0.0	-0.0	-0.0	0.0	-0.0
	1	-	22.58	B3LYP/6-31G(d,p)	0.2	-0.0	-0.0	0.0	0.2
	2	x, y, z	21.89	B3LYP/6-31G(d,p)	-0.0	-0.0	-0.0	0.0	-0.0
	2	x, y, z	24.96	B3LYP/6-31G(d,p)	0.0	-0.0	-0.0	0.0	0.0
	1	-	17.06	B3LYP/6-31G(d,p)	-0.2	-0.0	-0.1	0.0	-0.3
	1	-	31.03	B3LYP/6-31G(d,p)	0.1	-0.0	-0.0	0.0	0.1
	1	-	27.19	B3LYP/6-31G(d,p)	0.3	-0.0	-0.0	0.0	0.3
	1	-	10.92	B3LYP/6-31G(d,p)	-1.9	-0.2	-1.2	0.0	-3.2
	2	x, y, z	20.83	B3LYP/6-31G(d,p)	0.1	-0.0	-0.0	0.0	0.1
	2	x, y, z	12.98	B3LYP/6-31G(d,p)	1.4	-0.1	-0.5	0.0	1.0
	1	-	14.94	B3LYP/6-31G(d,p)	0.6	-0.1	-0.2	0.0	0.4
	2	x, y, z	11.80	B3LYP/6-31G(d,p)	-0.5	-0.1	-0.9	0.0	-1.4
	1	-	24.55	B3LYP/6-31G(d,p)	0.6	-0.0	-0.0	0.0	0.6
	2	x, y, z	19.65	B3LYP/6-31G(d,p)	-1.1	-0.0	-0.1	0.0	-1.2
	1	-	22.29	B3LYP/6-31G(d,p)	0.2	-0.0	-0.0	0.0	0.1
	1	-	28.16	B3LYP/6-31G(d,p)	-0.1	-0.0	-0.0	0.0	-0.1

	1	-	14.70	B3LYP/6-31G(d,p)	-1.3	-0.0	-0.3	0.0	-1.7
	2	x, y, z	14.56	B3LYP/6-31G(d,p)	-0.8	-0.1	-0.3	0.0	-1.2
	1	-	14.54	B3LYP/6-31G(d,p)	-0.1	-0.0	-0.2	0.0	-0.3
	2	x, y, z	24.05	B3LYP/6-31G(d,p)	0.2	-0.0	-0.0	0.0	0.2
	2	x, y, z	8.61	B3LYP/6-31G(d,p)	-1.9	-3.6	-15.3	17.2	-7.4
	1	-	21.97	B3LYP/6-31G(d,p)	0.2	-0.0	-0.0	0.0	0.1
	2	x, y, z	14.57	B3LYP/6-31G(d,p)	-1.3	-0.3	-0.8	0.0	-2.3
	2	x, y, z	29.14	B3LYP/6-31G(d,p)	-0.2	-0.0	-0.0	0.0	-0.2
	2	x, y, z	25.03	B3LYP/6-31G(d,p)	-0.7	-0.0	-0.0	0.0	-0.7
	1	-	24.25	B3LYP/6-31G(d,p)	-0.1	-0.0	-0.0	0.0	-0.2
	2	x, y, z	16.85	B3LYP/6-31G(d,p)	0.5	-0.0	-0.1	0.0	0.4
	1	-	21.78	B3LYP/6-31G(d,p)	-0.3	-0.0	-0.0	0.0	-0.3
	2	x, y, z	24.66	B3LYP/6-31G(d,p)	0.3	-0.0	-0.0	0.0	0.3
	2	x, y, z	17.70	B3LYP/6-31G(d,p)	0.5	-0.0	-0.1	0.0	0.5
	1	-	25.24	B3LYP/6-31G(d,p)	-0.1	-0.0	-0.0	0.0	-0.2
	2	x, y, z	24.42	B3LYP/6-31G(d,p)	0.3	-0.0	-0.0	0.0	0.3
	1	-	18.90	B3LYP/6-31G(d,p)	-0.4	-0.0	-0.0	0.0	-0.5
	2	x, y, z	21.76	B3LYP/6-31G(d,p)	0.0	-0.0	-0.0	0.0	-0.0
	1	-	26.40	B3LYP/6-31G(d,p)	0.1	-0.0	-0.0	0.0	0.1
	1	-	17.14	B3LYP/6-31G(d,p)	-0.5	-0.0	-0.1	0.0	-0.6
	2	x, y, z	21.69	B3LYP/6-31G(d,p)	0.3	-0.0	-0.0	0.0	0.3
	1	-	22.50	B3LYP/6-31G(d,p)	0.2	-0.0	-0.0	0.0	0.2
	2	x, y, z	23.45	B3LYP/6-31G(d,p)	0.1	-0.0	-0.0	0.0	0.0
	1	-	27.39	B3LYP/6-31G(d,p)	-0.1	-0.0	-0.0	0.0	-0.1

	2	x, y, z	28.89	B3LYP/6-31G(d,p)	-0.2	-0.0	-0.0	0.0	-0.2
	1	-	25.76	B3LYP/6-31G(d,p)	0.5	-0.0	-0.0	0.0	0.5
	1	-	14.68	B3LYP/6-31G(d,p)	1.4	-0.2	-0.4	0.0	1.0
	2	x, y, z	25.01	B3LYP/6-31G(d,p)	-0.5	-0.0	-0.0	0.0	-0.5
	2	x, y, z	21.73	B3LYP/6-31G(d,p)	-0.0	-0.0	-0.0	0.0	-0.1
	1	-	6.58	B3LYP/6-31G(d,p)	-6.8	-1.0	-35.7	19.2	-27.1
	2	x, y, z	12.92	B3LYP/6-31G(d,p)	1.0	-0.1	-0.6	0.0	0.5
	1	-	12.12	B3LYP/6-31G(d,p)	-4.6	-2.2	-5.6	0.0	-11.4
	2	x, y, z	11.73	B3LYP/6-31G(d,p)	0.5	-0.1	-0.7	0.0	-0.2
	1	-	22.95	B3LYP/6-31G(d,p)	1.0	-0.0	-0.0	0.0	1.1
	2	x, y, z	19.60	B3LYP/6-31G(d,p)	-0.8	-0.0	-0.0	0.0	-0.9
	2	x, y, z	30.15	B3LYP/6-31G(d,p)	-0.4	-0.0	-0.0	0.0	-0.4
	1	-	18.67	B3LYP/6-31G(d,p)	1.6	-0.1	-0.1	0.0	1.5
	1	-	8.24	B3LYP/6-31G(d,p)	-15.6	-4.5	-40.0	25.1	-39.2
	2	x, y, z	23.03	B3LYP/6-31G(d,p)	-0.2	-0.0	-0.0	0.0	-0.2
	2	x, y, z	11.75	B3LYP/6-31G(d,p)	-19.5	-7.5	-15.6	17.3	-29.1
	1	-	7.93	B3LYP/6-31G(d,p)	-15.6	-5.0	-28.6	19.0	-33.4
	1	-	18.27	B3LYP/6-31G(d,p)	0.2	-0.0	-0.1	0.0	0.1
	1	-	29.68	B3LYP/6-31G(d,p)	0.2	-0.0	-0.0	0.0	0.2
	2	x, y, z	23.49	B3LYP/6-31G(d,p)	-0.9	-0.0	-0.0	0.0	-1.0
	1	-	19.17	B3LYP/6-31G(d,p)	-0.0	-0.0	-0.0	0.0	-0.1
	1	-	15.93	B3LYP/6-31G(d,p)	-0.5	-0.0	-0.1	0.0	-0.6
	1	-	20.40	B3LYP/6-31G(d,p)	-0.4	-0.0	-0.0	0.0	-0.4
	2	x, y, z	24.88	B3LYP/6-31G(d,p)	-0.0	-0.0	-0.0	0.0	-0.0

	1	-	27.81	B3LYP/6-31G(d,p)	-0.1	-0.0	-0.0	0.0	-0.1
	2	x, y, z	25.83	B3LYP/6-31G(d,p)	0.2	-0.0	-0.0	0.0	0.2
	1	-	22.41	B3LYP/6-31G(d,p)	-0.3	-0.0	-0.0	0.0	-0.3
	1	-	20.94	B3LYP/6-31G(d,p)	-0.3	-0.0	-0.0	0.0	-0.3
	1	-	25.50	B3LYP/6-31G(d,p)	0.1	-0.0	-0.0	0.0	0.1
	1	-	27.14	B3LYP/6-31G(d,p)	0.3	-0.0	-0.0	0.0	0.3
	1	-	16.97	B3LYP/6-31G(d,p)	-0.0	-0.0	-0.1	0.0	-0.2
	1	-	10.75	B3LYP/6-31G(d,p)	-2.0	-0.1	-1.4	0.0	-3.4
	1	-	14.80	B3LYP/6-31G(d,p)	0.7	-0.1	-0.3	0.0	0.5
	1	-	24.46	B3LYP/6-31G(d,p)	0.6	-0.0	-0.0	0.0	0.6
	1	-	18.68	B3LYP/6-31G(d,p)	1.7	-0.1	-0.1	0.0	1.6
	1	-	30.09	B3LYP/6-31G(d,p)	0.4	-0.0	-0.0	0.0	0.4
	1	-	30.10	B3LYP/6-31G(d,p)	0.4	-0.0	-0.0	0.0	0.4
	1	-	8.24	B3LYP/6-31G(d,p)	-16.1	-4.5	-40.6	26.2	-39.6
	1	-	7.92	B3LYP/6-31G(d,p)	-16.9	-5.0	-29.7	21.2	-34.3
	1	-	18.26	B3LYP/6-31G(d,p)	0.3	-0.0	-0.1	0.0	0.2
	1	-	29.67	B3LYP/6-31G(d,p)	0.2	-0.0	-0.0	0.0	0.2
	1	-	27.51	B3LYP/6-31G(d,p)	0.2	-0.0	-0.0	0.0	0.2
	1	-	26.17	B3LYP/6-31G(d,p)	0.4	-0.0	-0.0	0.0	0.4
	1	-	17.18	B3LYP/6-31G(d,p)	0.3	-0.0	-0.1	0.0	0.3
	1	-	13.46	B3LYP/6-31G(d,p)	0.1	-0.0	-0.3	0.0	-0.2
	1	-	18.53	B3LYP/6-31G(d,p)	-0.4	-0.0	-0.1	0.0	-0.5
	1	-	27.95	B3LYP/6-31G(d,p)	-0.0	-0.0	-0.0	0.0	-0.0
	1	-	23.97	B3LYP/6-31G(d,p)	-0.0	-0.0	-0.0	0.0	-0.1

	1	-	25.07	B3LYP/6-31G(d,p)	-0.1	-0.0	-0.0	0.0	-0.2
	1	-	22.56	B3LYP/6-31G(d,p)	-0.2	-0.0	-0.0	0.0	-0.3
	1	-	30.94	B3LYP/6-31G(d,p)	0.1	-0.0	-0.0	0.0	0.1
	1	-	18.34	B3LYP/6-31G(d,p)	-0.5	-0.0	-0.0	0.0	-0.6
	1	-	20.96	B3LYP/6-31G(d,p)	0.1	-0.0	-0.0	0.0	0.1
	1	-	22.30	B3LYP/6-31G(d,p)	0.2	-0.0	-0.0	0.0	0.2
	1	-	14.70	B3LYP/6-31G(d,p)	-1.2	-0.0	-0.3	0.0	-1.5
	1	-	14.52	B3LYP/6-31G(d,p)	-0.3	-0.0	-0.2	0.0	-0.4
	1	-	21.94	B3LYP/6-31G(d,p)	0.1	-0.0	-0.0	0.0	0.1
	1	-	27.58	B3LYP/6-31G(d,p)	0.2	-0.0	-0.0	0.0	0.2
	1	-	19.26	B3LYP/6-31G(d,p)	-0.0	-0.0	-0.0	0.0	-0.0
	1	-	16.03	B3LYP/6-31G(d,p)	-0.5	-0.0	-0.1	0.0	-0.6
	1	-	20.47	B3LYP/6-31G(d,p)	-0.4	-0.0	-0.0	0.0	-0.4
	1	-	24.04	B3LYP/6-31G(d,p)	-0.0	-0.0	-0.0	0.0	-0.1
	1	-	25.13	B3LYP/6-31G(d,p)	-0.1	-0.0	-0.0	0.0	-0.2
	1	-	26.55	B3LYP/6-31G(d,p)	-0.2	-0.0	-0.0	0.0	-0.2
	1	-	28.18	B3LYP/6-31G(d,p)	-0.0	-0.0	-0.0	0.0	-0.0
	1	-	22.64	B3LYP/6-31G(d,p)	-0.3	-0.0	-0.0	0.0	-0.3
	1	-	22.52	B3LYP/6-31G(d,p)	-0.2	-0.0	-0.0	0.0	-0.2
	1	-	24.39	B3LYP/6-31G(d,p)	-0.1	-0.0	-0.0	0.0	-0.1
	1	-	21.93	B3LYP/6-31G(d,p)	-0.3	-0.0	-0.0	0.0	-0.3
	1	-	25.35	B3LYP/6-31G(d,p)	-0.2	-0.0	-0.0	0.0	-0.2

Table S10. Lattice energy of GSF-nBuAc desolvated calculated for GSF molecule B at 20 Å cluster of molecules using B3LYP/6-31G(d,p) wavefunction; the total intermolecular interaction energy for a given molecule is the sum of the electrostatic, polarization, dispersion, and exchange-repulsion components with scale factors of 1.057, 0.740, 0.871, and 0.618, respectively.

	N	Symop	R	Electron Density	E_ele	E_pol	E_dis	E_rep	E_tot
	1	-	22.63	B3LYP/6-31G(d,p)	-0.3	-0.0	-0.0	0.0	-0.4
	1	-	21.19	B3LYP/6-31G(d,p)	-0.3	-0.0	-0.0	0.0	-0.3
	1	-	25.73	B3LYP/6-31G(d,p)	0.1	-0.0	-0.0	0.0	0.1
	0	x, y, z	24.82	B3LYP/6-31G(d,p)	0.2	-0.0	-0.0	0.0	0.2
	1	-	22.70	B3LYP/6-31G(d,p)	-0.3	-0.0	-0.0	0.0	-0.3
	1	-	18.53	B3LYP/6-31G(d,p)	-0.5	-0.0	-0.0	0.0	-0.5
	0	x, y, z	17.84	B3LYP/6-31G(d,p)	0.4	-0.0	-0.0	0.0	0.4
	0	x, y, z	24.24	B3LYP/6-31G(d,p)	0.3	-0.0	-0.0	0.0	0.3
	1	-	26.75	B3LYP/6-31G(d,p)	-0.2	-0.0	-0.0	0.0	-0.2
	1	-	21.15	B3LYP/6-31G(d,p)	0.1	-0.0	-0.0	0.0	0.1
	0	x, y, z	17.01	B3LYP/6-31G(d,p)	0.5	-0.0	-0.1	0.0	0.5
	0	x, y, z	28.89	B3LYP/6-31G(d,p)	-0.0	-0.0	-0.0	0.0	-0.0
	0	x, y, z	23.16	B3LYP/6-31G(d,p)	-0.3	-0.0	-0.0	0.0	-0.3
	1	-	22.64	B3LYP/6-31G(d,p)	-0.3	-0.0	-0.0	0.0	-0.4
	0	x, y, z	25.83	B3LYP/6-31G(d,p)	0.3	-0.0	-0.0	0.0	0.3
	1	-	22.54	B3LYP/6-31G(d,p)	-0.1	-0.0	-0.0	0.0	-0.1
	0	x, y, z	17.22	B3LYP/6-31G(d,p)	0.9	-0.0	-0.1	0.0	0.8
	0	x, y, z	20.85	B3LYP/6-31G(d,p)	0.2	-0.0	-0.0	0.0	0.2
	0	x, y, z	25.95	B3LYP/6-31G(d,p)	0.2	-0.0	-0.0	0.0	0.2

	1	-	18.99	B3LYP/6-31G(d,p)	-0.4	-0.0	-0.0	0.0	-0.5
	1	-	26.46	B3LYP/6-31G(d,p)	0.0	-0.0	-0.0	0.0	0.0
	0	x, y, z	21.85	B3LYP/6-31G(d,p)	0.3	-0.0	-0.0	0.0	0.3
	1	-	17.24	B3LYP/6-31G(d,p)	-0.5	-0.0	-0.1	0.0	-0.6
	0	x, y, z	20.00	B3LYP/6-31G(d,p)	0.4	-0.0	-0.0	0.0	0.4
	0	x, y, z	23.60	B3LYP/6-31G(d,p)	0.0	-0.0	-0.0	0.0	-0.0
	1	-	22.58	B3LYP/6-31G(d,p)	0.2	-0.0	-0.0	0.0	0.2
	0	x, y, z	21.89	B3LYP/6-31G(d,p)	-0.0	-0.0	-0.0	0.0	-0.0
	0	x, y, z	24.96	B3LYP/6-31G(d,p)	0.0	-0.0	-0.0	0.0	0.0
	1	-	17.06	B3LYP/6-31G(d,p)	-0.2	-0.0	-0.1	0.0	-0.3
	1	-	31.03	B3LYP/6-31G(d,p)	0.1	-0.0	-0.0	0.0	0.1
	1	-	27.19	B3LYP/6-31G(d,p)	0.3	-0.0	-0.0	0.0	0.3
	1	-	10.92	B3LYP/6-31G(d,p)	-1.9	-0.2	-1.2	0.0	-3.2
	0	x, y, z	20.83	B3LYP/6-31G(d,p)	0.1	-0.0	-0.0	0.0	0.1
	0	x, y, z	12.98	B3LYP/6-31G(d,p)	1.4	-0.1	-0.5	0.0	1.0
	1	-	14.94	B3LYP/6-31G(d,p)	0.6	-0.1	-0.2	0.0	0.4
	0	x, y, z	11.80	B3LYP/6-31G(d,p)	-0.5	-0.1	-0.9	0.0	-1.4
	1	-	24.55	B3LYP/6-31G(d,p)	0.6	-0.0	-0.0	0.0	0.6
	0	x, y, z	19.65	B3LYP/6-31G(d,p)	-1.1	-0.0	-0.1	0.0	-1.2
	1	-	22.29	B3LYP/6-31G(d,p)	0.2	-0.0	-0.0	0.0	0.1
	1	-	28.16	B3LYP/6-31G(d,p)	-0.1	-0.0	-0.0	0.0	-0.1
	1	-	14.70	B3LYP/6-31G(d,p)	-1.3	-0.0	-0.3	0.0	-1.7
	0	x, y, z	14.56	B3LYP/6-31G(d,p)	-0.8	-0.1	-0.3	0.0	-1.2
	1	-	14.54	B3LYP/6-31G(d,p)	-0.1	-0.0	-0.2	0.0	-0.3

	0	x, y, z	24.05	B3LYP/6-31G(d,p)	0.2	-0.0	-0.0	0.0	0.2
	0	x, y, z	8.61	B3LYP/6-31G(d,p)	-1.9	-3.6	-15.3	17.2	-7.4
	1	-	21.97	B3LYP/6-31G(d,p)	0.2	-0.0	-0.0	0.0	0.1
	0	x, y, z	14.57	B3LYP/6-31G(d,p)	-1.3	-0.3	-0.8	0.0	-2.3
	0	x, y, z	29.14	B3LYP/6-31G(d,p)	-0.2	-0.0	-0.0	0.0	-0.2
	0	x, y, z	25.03	B3LYP/6-31G(d,p)	-0.7	-0.0	-0.0	0.0	-0.7
	1	-	24.25	B3LYP/6-31G(d,p)	-0.1	-0.0	-0.0	0.0	-0.2
	0	x, y, z	16.85	B3LYP/6-31G(d,p)	0.5	-0.0	-0.1	0.0	0.4
	1	-	21.78	B3LYP/6-31G(d,p)	-0.3	-0.0	-0.0	0.0	-0.3
	0	x, y, z	24.66	B3LYP/6-31G(d,p)	0.3	-0.0	-0.0	0.0	0.3
	0	x, y, z	17.70	B3LYP/6-31G(d,p)	0.5	-0.0	-0.1	0.0	0.5
	1	-	25.24	B3LYP/6-31G(d,p)	-0.1	-0.0	-0.0	0.0	-0.2
	0	x, y, z	24.42	B3LYP/6-31G(d,p)	0.3	-0.0	-0.0	0.0	0.3
	1	-	18.90	B3LYP/6-31G(d,p)	-0.4	-0.0	-0.0	0.0	-0.5
	0	x, y, z	21.76	B3LYP/6-31G(d,p)	0.0	-0.0	-0.0	0.0	-0.0
	1	-	26.40	B3LYP/6-31G(d,p)	0.1	-0.0	-0.0	0.0	0.1
	1	-	17.14	B3LYP/6-31G(d,p)	-0.5	-0.0	-0.1	0.0	-0.6
	0	x, y, z	21.69	B3LYP/6-31G(d,p)	0.3	-0.0	-0.0	0.0	0.3
	1	-	22.50	B3LYP/6-31G(d,p)	0.2	-0.0	-0.0	0.0	0.2
	0	x, y, z	23.45	B3LYP/6-31G(d,p)	0.1	-0.0	-0.0	0.0	0.0
	1	-	27.39	B3LYP/6-31G(d,p)	-0.1	-0.0	-0.0	0.0	-0.1
	0	x, y, z	28.89	B3LYP/6-31G(d,p)	-0.2	-0.0	-0.0	0.0	-0.2
	1	-	25.76	B3LYP/6-31G(d,p)	0.5	-0.0	-0.0	0.0	0.5
	1	-	14.68	B3LYP/6-31G(d,p)	1.4	-0.2	-0.4	0.0	1.0

	0	x, y, z	25.01	B3LYP/6-31G(d,p)	-0.5	-0.0	-0.0	0.0	-0.5
	0	x, y, z	21.73	B3LYP/6-31G(d,p)	-0.0	-0.0	-0.0	0.0	-0.1
	1	-	6.58	B3LYP/6-31G(d,p)	-6.8	-1.0	-35.7	19.2	-27.1
	0	x, y, z	12.92	B3LYP/6-31G(d,p)	1.0	-0.1	-0.6	0.0	0.5
	1	-	12.12	B3LYP/6-31G(d,p)	-4.6	-2.2	-5.6	0.0	-11.4
	0	x, y, z	11.73	B3LYP/6-31G(d,p)	0.5	-0.1	-0.7	0.0	-0.2
	1	-	22.95	B3LYP/6-31G(d,p)	1.0	-0.0	-0.0	0.0	1.1
	0	x, y, z	19.60	B3LYP/6-31G(d,p)	-0.8	-0.0	-0.0	0.0	-0.9
	0	x, y, z	30.15	B3LYP/6-31G(d,p)	-0.4	-0.0	-0.0	0.0	-0.4
	1	-	18.67	B3LYP/6-31G(d,p)	1.6	-0.1	-0.1	0.0	1.5
	1	-	8.24	B3LYP/6-31G(d,p)	-15.6	-4.5	-40.0	25.1	-39.2
	0	x, y, z	23.03	B3LYP/6-31G(d,p)	-0.2	-0.0	-0.0	0.0	-0.2
	0	x, y, z	11.75	B3LYP/6-31G(d,p)	-19.5	-7.5	-15.6	17.3	-29.1
	1	-	7.93	B3LYP/6-31G(d,p)	-15.6	-5.0	-28.6	19.0	-33.4
	1	-	18.27	B3LYP/6-31G(d,p)	0.2	-0.0	-0.1	0.0	0.1
	1	-	29.68	B3LYP/6-31G(d,p)	0.2	-0.0	-0.0	0.0	0.2
	0	x, y, z	23.49	B3LYP/6-31G(d,p)	-0.9	-0.0	-0.0	0.0	-1.0
	1	-	19.17	B3LYP/6-31G(d,p)	-0.0	-0.0	-0.0	0.0	-0.1
	1	-	15.93	B3LYP/6-31G(d,p)	-0.5	-0.0	-0.1	0.0	-0.6
	1	-	20.40	B3LYP/6-31G(d,p)	-0.4	-0.0	-0.0	0.0	-0.4
	0	x, y, z	24.88	B3LYP/6-31G(d,p)	-0.0	-0.0	-0.0	0.0	-0.0
	1	-	27.81	B3LYP/6-31G(d,p)	-0.1	-0.0	-0.0	0.0	-0.1
	0	x, y, z	25.83	B3LYP/6-31G(d,p)	0.2	-0.0	-0.0	0.0	0.2
	1	-	22.41	B3LYP/6-31G(d,p)	-0.3	-0.0	-0.0	0.0	-0.3

	1	-	20.94	B3LYP/6-31G(d,p)	-0.3	-0.0	-0.0	0.0	-0.3
	1	-	25.50	B3LYP/6-31G(d,p)	0.1	-0.0	-0.0	0.0	0.1
	1	-	27.14	B3LYP/6-31G(d,p)	0.3	-0.0	-0.0	0.0	0.3
	1	-	16.97	B3LYP/6-31G(d,p)	-0.0	-0.0	-0.1	0.0	-0.2
	1	-	10.75	B3LYP/6-31G(d,p)	-2.0	-0.1	-1.4	0.0	-3.4
	1	-	14.80	B3LYP/6-31G(d,p)	0.7	-0.1	-0.3	0.0	0.5
	1	-	24.46	B3LYP/6-31G(d,p)	0.6	-0.0	-0.0	0.0	0.6
	1	-	18.68	B3LYP/6-31G(d,p)	1.7	-0.1	-0.1	0.0	1.6
	1	-	30.09	B3LYP/6-31G(d,p)	0.4	-0.0	-0.0	0.0	0.4
	1	-	30.10	B3LYP/6-31G(d,p)	0.4	-0.0	-0.0	0.0	0.4
	1	-	8.24	B3LYP/6-31G(d,p)	-16.1	-4.5	-40.6	26.2	-39.6
	1	-	7.92	B3LYP/6-31G(d,p)	-16.9	-5.0	-29.7	21.2	-34.3
	1	-	18.26	B3LYP/6-31G(d,p)	0.3	-0.0	-0.1	0.0	0.2
	1	-	29.67	B3LYP/6-31G(d,p)	0.2	-0.0	-0.0	0.0	0.2
	1	-	27.51	B3LYP/6-31G(d,p)	0.2	-0.0	-0.0	0.0	0.2
	1	-	26.17	B3LYP/6-31G(d,p)	0.4	-0.0	-0.0	0.0	0.4
	1	-	17.18	B3LYP/6-31G(d,p)	0.3	-0.0	-0.1	0.0	0.3
	1	-	13.46	B3LYP/6-31G(d,p)	0.1	-0.0	-0.3	0.0	-0.2
	1	-	18.53	B3LYP/6-31G(d,p)	-0.4	-0.0	-0.1	0.0	-0.5
	1	-	27.95	B3LYP/6-31G(d,p)	-0.0	-0.0	-0.0	0.0	-0.0
	1	-	23.97	B3LYP/6-31G(d,p)	-0.0	-0.0	-0.0	0.0	-0.1
	1	-	25.07	B3LYP/6-31G(d,p)	-0.1	-0.0	-0.0	0.0	-0.2
	1	-	22.56	B3LYP/6-31G(d,p)	-0.2	-0.0	-0.0	0.0	-0.3
	1	-	30.94	B3LYP/6-31G(d,p)	0.1	-0.0	-0.0	0.0	0.1

	1	-	18.34	B3LYP/6-31G(d,p)	-0.5	-0.0	-0.0	0.0	-0.6
	1	-	20.96	B3LYP/6-31G(d,p)	0.1	-0.0	-0.0	0.0	0.1
	1	-	22.30	B3LYP/6-31G(d,p)	0.2	-0.0	-0.0	0.0	0.2
	1	-	14.70	B3LYP/6-31G(d,p)	-1.2	-0.0	-0.3	0.0	-1.5
	1	-	14.52	B3LYP/6-31G(d,p)	-0.3	-0.0	-0.2	0.0	-0.4
	1	-	21.94	B3LYP/6-31G(d,p)	0.1	-0.0	-0.0	0.0	0.1
	1	-	27.58	B3LYP/6-31G(d,p)	0.2	-0.0	-0.0	0.0	0.2
	1	-	19.26	B3LYP/6-31G(d,p)	-0.0	-0.0	-0.0	0.0	-0.0
	1	-	16.03	B3LYP/6-31G(d,p)	-0.5	-0.0	-0.1	0.0	-0.6
	1	-	20.47	B3LYP/6-31G(d,p)	-0.4	-0.0	-0.0	0.0	-0.4
	1	-	24.04	B3LYP/6-31G(d,p)	-0.0	-0.0	-0.0	0.0	-0.1
	1	-	25.13	B3LYP/6-31G(d,p)	-0.1	-0.0	-0.0	0.0	-0.2
	1	-	26.55	B3LYP/6-31G(d,p)	-0.2	-0.0	-0.0	0.0	-0.2
	1	-	28.18	B3LYP/6-31G(d,p)	-0.0	-0.0	-0.0	0.0	-0.0
	1	-	22.64	B3LYP/6-31G(d,p)	-0.3	-0.0	-0.0	0.0	-0.3
	1	-	22.52	B3LYP/6-31G(d,p)	-0.2	-0.0	-0.0	0.0	-0.2
	1	-	24.39	B3LYP/6-31G(d,p)	-0.1	-0.0	-0.0	0.0	-0.1
	1	-	21.93	B3LYP/6-31G(d,p)	-0.3	-0.0	-0.0	0.0	-0.3
	1	-	25.35	B3LYP/6-31G(d,p)	-0.2	-0.0	-0.0	0.0	-0.2
	2	x, y, z	21.85	B3LYP/6-31G(d,p)	0.2	-0.0	-0.0	0.0	0.2
	2	x, y, z	25.95	B3LYP/6-31G(d,p)	0.1	-0.0	-0.0	0.0	0.1
	2	x, y, z	23.60	B3LYP/6-31G(d,p)	0.1	-0.0	-0.0	0.0	0.1
	2	x, y, z	17.84	B3LYP/6-31G(d,p)	0.3	-0.0	-0.1	0.0	0.3
	2	x, y, z	24.96	B3LYP/6-31G(d,p)	-0.0	-0.0	-0.0	0.0	-0.0

	2	x, y, z	24.82	B3LYP/6-31G(d,p)	0.2	-0.0	-0.0	0.0	0.2
	2	x, y, z	17.01	B3LYP/6-31G(d,p)	0.3	-0.0	-0.1	0.0	0.3
	2	x, y, z	24.24	B3LYP/6-31G(d,p)	0.2	-0.0	-0.0	0.0	0.2
	2	x, y, z	23.16	B3LYP/6-31G(d,p)	-0.2	-0.0	-0.0	0.0	-0.2
	2	x, y, z	20.83	B3LYP/6-31G(d,p)	0.2	-0.0	-0.0	0.0	0.2
	2	x, y, z	17.22	B3LYP/6-31G(d,p)	0.7	-0.0	-0.1	0.0	0.7
	2	x, y, z	25.83	B3LYP/6-31G(d,p)	0.2	-0.0	-0.0	0.0	0.2
	2	x, y, z	20.85	B3LYP/6-31G(d,p)	0.0	-0.0	-0.0	0.0	-0.0
	2	x, y, z	24.66	B3LYP/6-31G(d,p)	0.2	-0.0	-0.0	0.0	0.2
	2	x, y, z	24.42	B3LYP/6-31G(d,p)	0.2	-0.0	-0.0	0.0	0.2
	2	x, y, z	20.00	B3LYP/6-31G(d,p)	0.3	-0.0	-0.0	0.0	0.3
	2	x, y, z	21.89	B3LYP/6-31G(d,p)	0.0	-0.0	-0.0	0.0	0.0
	2	x, y, z	21.76	B3LYP/6-31G(d,p)	-0.0	-0.0	-0.0	0.0	-0.1
	2	x, y, z	12.98	B3LYP/6-31G(d,p)	0.4	-0.1	-0.6	0.0	-0.1
	2	x, y, z	11.80	B3LYP/6-31G(d,p)	0.4	-0.0	-0.7	0.0	-0.2
	2	x, y, z	19.65	B3LYP/6-31G(d,p)	-0.6	-0.0	-0.0	0.0	-0.7
	2	x, y, z	29.11	B3LYP/6-31G(d,p)	-0.1	-0.0	-0.0	0.0	-0.2
	2	x, y, z	25.01	B3LYP/6-31G(d,p)	-0.5	-0.0	-0.0	0.0	-0.6
	2	x, y, z	14.56	B3LYP/6-31G(d,p)	-0.5	-0.2	-0.8	0.0	-1.5
	2	x, y, z	8.61	B3LYP/6-31G(d,p)	-2.0	-3.8	-15.2	17.1	-7.5
	2	x, y, z	14.57	B3LYP/6-31G(d,p)	-1.1	-0.1	-0.3	0.0	-1.5
	2	x, y, z	25.03	B3LYP/6-31G(d,p)	-0.4	-0.0	-0.0	0.0	-0.4
	2	x, y, z	23.03	B3LYP/6-31G(d,p)	-0.2	-0.0	-0.0	0.0	-0.2
	2	x, y, z	16.85	B3LYP/6-31G(d,p)	0.5	-0.0	-0.1	0.0	0.4

	2	x, y, z	28.72	B3LYP/6-31G(d,p)	0.0	-0.0	-0.0	0.0	0.0
	2	x, y, z	24.05	B3LYP/6-31G(d,p)	0.2	-0.0	-0.0	0.0	0.2
	2	x, y, z	17.70	B3LYP/6-31G(d,p)	0.2	-0.0	-0.1	0.0	0.2
	2	x, y, z	24.88	B3LYP/6-31G(d,p)	-0.0	-0.0	-0.0	0.0	-0.0
	2	x, y, z	25.83	B3LYP/6-31G(d,p)	0.1	-0.0	-0.0	0.0	0.1
	2	x, y, z	21.69	B3LYP/6-31G(d,p)	0.2	-0.0	-0.0	0.0	0.2
	2	x, y, z	23.45	B3LYP/6-31G(d,p)	0.0	-0.0	-0.0	0.0	0.0
	2	x, y, z	28.89	B3LYP/6-31G(d,p)	-0.1	-0.0	-0.0	0.0	-0.2
	2	x, y, z	21.73	B3LYP/6-31G(d,p)	0.0	-0.0	-0.0	0.0	-0.0
	2	x, y, z	12.92	B3LYP/6-31G(d,p)	0.9	-0.1	-0.5	0.0	0.4
	2	x, y, z	11.73	B3LYP/6-31G(d,p)	-0.3	-0.1	-0.9	0.0	-1.2
	2	x, y, z	19.60	B3LYP/6-31G(d,p)	-0.8	-0.0	-0.1	0.0	-0.9
	2	x, y, z	30.11	B3LYP/6-31G(d,p)	-0.3	-0.0	-0.0	0.0	-0.3
	2	x, y, z	23.49	B3LYP/6-31G(d,p)	-0.7	-0.0	-0.0	0.0	-0.8
	2	x, y, z	11.75	B3LYP/6-31G(d,p)	-17.4	-6.4	-16.1	17.9	-26.2

REFERENCES

[1] J. F. Grove and J. C. McGowan, "Identity of Griseofulvin and 'Curling-factor,'" *Nature*, vol. 160, pp. 574–574, 1947.

[2] A. Mahieu, J. Willart, E. Dudognon, "On the Polymorphism of Griseofulvin: Identification of Two Additional Polymorphs," *J. Pharm. Sci*., vol. 102, no. 7, pp. 2271–2280, 2013, doi: 10.1002/jps.

[3] X. Ou, S. Li, Y. Chen, H. Rong, A. Li, and M. Lu, "Polymorphism in Griseofulvin: New Story between an Old Drug and Polyethylene Glycol," *Cryst. Growth Des*., vol. 22, pp. 3778-3785, 2022, doi: 10.1021/acs.cgd.2c00156.

[4] G. Malmros, A. Wagner, and L. Maron, “(2S.6’R)-7-CHLORO 2’,4,6-TRIMETHOXY-6’-METHYL-SPIRO(BENZOFURAN-2(3H), 2-(2’)CYCLOHEXENE-3,4’-DIONE, C17H17CLO6,” *Cryst. Struct. Commun*., vol. 6, pp. 463 - 470, 1977.

[5] Y. Su, J. Xu, Q. Shi, L. Yu, and T. Cai, “Polymorphism of griseofulvin: Concomitant crystallization from the melt and a single crystal structure of a metastable polymorph with anomalously large thermal expansion,” *Chem. Commun*., vol. 54, no. 4, pp. 358–361, 2018, doi: 10.1039/c7cc07744k.

[6] X. Ou, X. Li, H. Rong, L. Yu, and M. Lu, “A general method for cultivating single crystals from melt microdroplets,” *Chem. Commun*., vol. 56, no. 69, pp. 9950–9953, 2020, doi: 10.1039/d0cc03157g.

[7] Z. Dauter, A. Hempel, M. Bogucka-Ledochowska, E. Borowski, and W. Dreissig, “Griseofulvin-benzene solvate,” *Eur. Cryst. Meeting*, vol. 5, p. 46, 1979.

[8] K. C. Cheng, E. L. I. Shefter, T. Srikrishnan, and U. S. P. A. Laboratories, “Crystal structure analysis of the desolvation chloroform solvate of griseofulvin,” *Inter. Jour. Pharm*., vol. 2, pp. 81–89, 1979.

[9] S. Aitipamula, P. S. Chow, and R. B. H. Tan, “Solvates of the antifungal drug griseofulvin: Structural, thermochemical and conformational analysis,” *Acta Crystallogr*. Sect. B Struct. Sci. Cryst. Eng. Mater., vol. 70, no. 1, pp. 54–62, 2014, doi: 10.1107/S2052520613026711.

[10] K. Shirotani, E. Suzuki, and Y. Morita, “Solvate Formation of Griseofulvin with Alkyl Halide and Alkyl Dihalides,” *Chem. Pharm. Bull.,* no. 10, pp. 4045–4054, 1988.

[11] H. Chen, C. Wang, and C. C. Sun, “Profoundly Improved Plasticity and Tabletability of Griseofulvin by in Situ Solvation and Desolvation during Spherical Crystallization,” *Cryst. Growth Des*., vol. 19, no. 4, pp. 2350–2357, 2019, doi: 10.1021/acs.cgd.9b00053.
